# Regulation of Wnt/β‐catenin signalling by tankyrase‐dependent poly(ADP‐ribosyl)ation and scaffolding

**DOI:** 10.1111/bph.14038

**Published:** 2017-11-05

**Authors:** Laura Mariotti, Katie Pollock, Sebastian Guettler

**Affiliations:** ^1^ Division of Structural Biology The Institute of Cancer Research London UK; ^2^ Division of Cancer Biology The Institute of Cancer Research London UK; ^3^ Division of Cancer Therapeutics The Institute of Cancer Research London UK

## Abstract

The Wnt/β‐catenin signalling pathway is pivotal for stem cell function and the control of cellular differentiation, both during embryonic development and tissue homeostasis in adults. Its activity is carefully controlled through the concerted interactions of concentration‐limited pathway components and a wide range of post‐translational modifications, including phosphorylation, ubiquitylation, sumoylation, poly(ADP‐ribosyl)ation (PARylation) and acetylation. Regulation of Wnt/β‐catenin signalling by PARylation was discovered relatively recently. The PARP tankyrase PARylates AXIN1/2, an essential central scaffolding protein in the β‐catenin destruction complex, and targets it for degradation, thereby fine‐tuning the responsiveness of cells to the Wnt signal. The past few years have not only seen much progress in our understanding of the molecular mechanisms by which PARylation controls the pathway but also witnessed the successful development of tankyrase inhibitors as tool compounds and promising agents for the therapy of Wnt‐dependent dysfunctions, including colorectal cancer. Recent work has hinted at more complex roles of tankyrase in Wnt/β‐catenin signalling as well as challenges and opportunities in the development of tankyrase inhibitors. Here we review some of the latest advances in our understanding of tankyrase function in the pathway and efforts to modulate tankyrase activity to re‐tune Wnt/β‐catenin signalling in colorectal cancer cells.

**Linked Articles:**

This article is part of a themed section on WNT Signalling: Mechanisms and Therapeutic Opportunities. To view the other articles in this section visit http://onlinelibrary.wiley.com/doi/10.1111/bph.v174.24/issuetoc

**Abbreviations**
APC: adenomatous polyposis coli
ARC: ankyrin repeat cluster
ARTD: Diphtheria‐toxin‐like ADP‐ribosyltransferase
AXIN: axis inhibition protein
B9L: B‐cell CLL/lymphoma 9‐like protein
CRC: colorectal cancer
DKK1: Dickkopf‐related protein 1
DVL: Dishevelled
FRAP: fluorescence recovery after photobleaching
GSK3: glycogen synthase kinase 3
ISC: intestinal stem cell
LRP5/6: LDL receptor‐related protein 5/6
NAD^+^: nicotinamide adenine dinucleotide
PARdU: PAR‐dependent ubiquitylation
RING: really interesting new gene
RNF: RING finger
SAM: sterile α motif
TBM: tankyrase‐binding motif
TNKS/TNKS2: tankyrase
TNKSi: tankyrase inhibitor
V5: epitope tag derived from the P and V proteins of paramyxovirus and simian virus 5, respectively
Wg/WNT: Wingless and its vertebrate orthologue
WWE: domain named after a motif containing two conserved Trp (W) residues and one conserved Glu (E)
β‐TRCP: β‐transducin repeats‐containing protein

## Regulation of Wnt/β‐catenin signalling by tankyrase‐dependent AXIN poly(ADP‐ribosyl)ation – an overview

**Figure 1:**
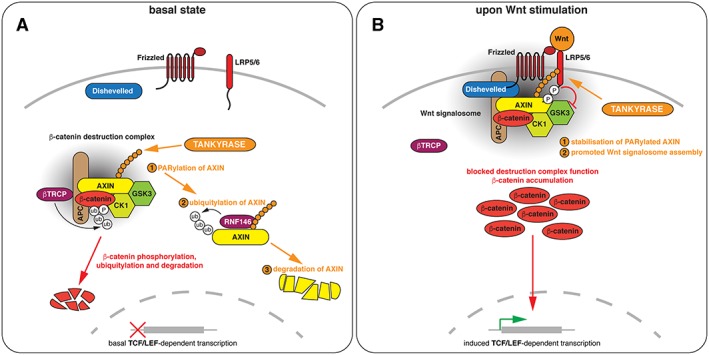
Roles of tankyrase‐dependent poly(ADP‐ribosyl)ation (PARylation) in Wnt/β‐catenin signalling. (A) Under basal Wnt/β‐catenin signalling conditions, PARylation by tankyrase limits the levels of AXIN. Following PARylation, AXIN is ubiquitylated by RNF146 and targeted for proteasomal degradation. (B) Upon Wnt stimulation, PARylated AXIN is stabilized. PARylation facilitates AXIN interaction with LRP5/6 in Wnt signalosomes. Note that AXIN, Dishevelled and tankyrase polymerize and APC dimerizes, and that this is a mechanistically important aspect of the dynamic signalling complexes (Fiedler *et al.,* 2011; Kunttas‐Tatli *et al.,* 2014; Mariotti *et al.,* 2016). For simplicity, proteins are shown as monomers; higher‐order stoichiometry and multivalency are not reflected in the diagrams and nomenclature does not consider multiple paralogues of pathway components.

The Wnt/β‐catenin signalling pathway plays key roles during embryonic development, tissue homeostasis and regeneration (see Clevers and Nusse, 2012; and Clevers *et al.,* 2014). Central to the pathway is the β‐catenin destruction complex, which tightly controls the levels of nuclear, transcriptionally active β‐catenin (see Stamos and Weis, 2013). Dysregulation of β‐catenin destruction complex function underlies the vast majority of colorectal cancers (CRCs) and other conditions such as fibrosis, neurodegeneration and osteoporosis (see Clevers and Nusse, 2012; and Kahn, 2014). AXIN (AXIN1/AXIN2), the central scaffold of the destruction complex, directly binds all its core components: the scaffolding protein adenomatous polyposis coli (APC), the kinases glycogen synthase kinase 3 (GSK3) and casein kinase 1 and β‐catenin (see Stamos and Weis, 2013) (Figure 1A). (AXIN1 and AXIN2 will be referred to collectively as AXIN where the discussed aspects apply to both.) The complex enables the phosphorylation of β‐catenin at a phosphodegron to prime it for ubiquitylation and subsequent degradation by the proteasome (see Stamos and Weis, 2013). AXIN is thought to be the concentration‐limiting component of the complex (Salic *et al.,* 2000; Lee *et al.,* 2003). Therefore, controlling its abundance is an effective way to regulate β‐catenin destruction. Wnt/β‐catenin signalling is regulated by a wide range of post‐translational modifications (see Gao *et al.,* 2014). The discovery of a regulatory role of tankyrase in Wnt/β‐catenin signalling sparked much excitement given the limited number of known targetable enzymes in the pathway (Huang *et al.,* 2009). Tankyrase, with two human paralogues (TNKS and TNKS2; Tnks and Tnks2 in other species discussed; from here on simply referred to as ‘tankyrase’ where concepts apply to both tankyrases), is a PARP, and as such catalyses the attachment of PAR chains onto its substrates (see Gibson and Kraus, 2012; and Haikarainen *et al.,* 2014a). In addition to its auto‐PARylation activity, tankyrase binds and PARylates AXIN. In turn, PARylation activates the PAR‐dependent E3 ubiquitin ligase really interesting new gene (RING) finger (RNF)146/Iduna, which then ubiquitylates AXIN, tankyrase and itself, targeting the entire complex for proteasomal degradation (Callow *et al.,* 2011; Zhang *et al.,* 2011) (Figure 1A). This process, known as PAR‐dependent ubiquitylation (PARdU) (DaRosa *et al.,* 2015), is thought to occur constitutively and tune the receptiveness of cells to Wnt stimuli by limiting destruction complex formation (Wang *et al.,* 2016b). A tankyrase‐associated ubiquitin‐specific protease (USP25) can de‐ubiquitylate tankyrase, thereby stabilizing it and supporting PARdU of AXIN (Xu *et al.,* 2017a). Recent studies point toward another role of tankyrase, namely in promoting the formation of active, membrane‐localized Wnt signalosomes upon Wnt stimulation (Yang *et al.,* 2016; Wang *et al.,* 2016a) (Figure 1B). Furthermore, additional tankyrase interactors in the Wnt/β‐catenin pathway, other than AXIN, are emerging (Croy *et al.,* 2016). Structure–function studies are providing a detailed picture of the molecular mechanisms by which tankyrase controls Wnt/β‐catenin signalling and are revealing non‐catalytic scaffolding roles of tankyrase (Guettler *et al.,* 2011; Morrone *et al.,* 2012; DaRosa *et al.,* 2015, 2016; Eisemann *et al.,* 2016; Mariotti *et al.,* 2016; Riccio *et al.,* 2016; Xu *et al.,* 2017a). Conserved functions of tankyrase in the Wnt/β‐catenin pathway are being increasingly appreciated from studies in *Drosophila* and human CRC cell lines (Lau *et al.,* 2013; de la Roche *et al.,* 2014; Yang *et al.,* 2016; Wang *et al.,* 2016b, 2016c). Recently developed tankyrase‐specific catalytic inhibitors are serving as tool compounds and promising preclinical leads for the treatment of CRC and other Wnt‐dependent conditions (Lau *et al.,* 2013; see Haikarainen *et al.,* 2014a). Here, we discuss a selection of recent insights into the roles of tankyrase in the Wnt/β‐catenin signalling pathway. In terms of potential therapeutic applications, we will focus on CRC, given its high incidence and the importance of the Wnt/β‐catenin pathway in its emergence.

## Tankyrase as a scaffold in Wnt/β‐catenin signalling – a structural perspective

**Figure 2:**
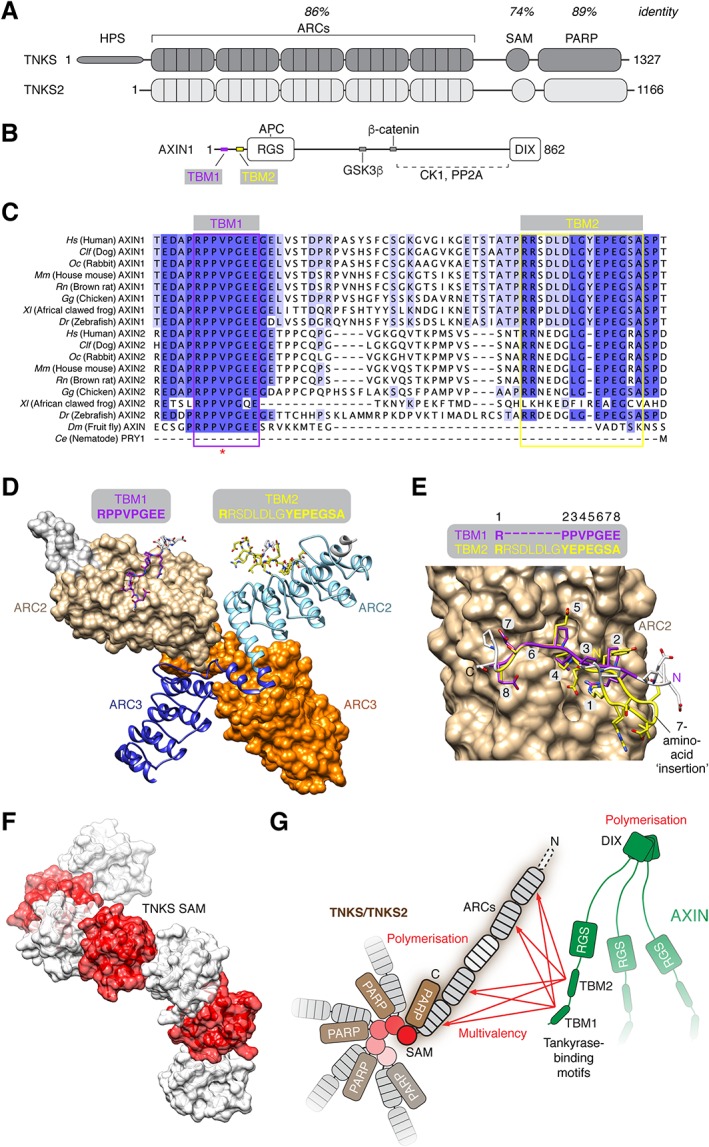
Scaffolding functions of tankyrase. (A) Domain organization of human TNKS and TNKS2. HPS, N‐terminal extension containing homopolymeric stretches of His, Pro and Ser; ARCs, ankyrin repeat clusters; SAM, sterile α motif domain; PARP, catalytic domain. The percentage identity of amino acids between TNKS and TNKS2 is specified for the indicated functional domains. (B) Domain organization of AXIN1. TBM, tankyrase‐binding motif; RGS, regulator of G‐protein signalling domain; DIX, polymerizing domain present in Dishevelled and AXIN (sometimes referred to as DAX domain in AXIN). Binding sites for other β‐catenin destruction complex components are indicated. (C) Multiple sequence alignment of AXIN orthologues/paralogues from the indicated species, coloured by percentage identity. The TBMs are indicated. Note that *Drosophila* Axin lacks the second TBM. The red asterisk denotes a V26D mutation identified in murine Axin2 (Qian *et al.,* 2011). (D) Structural (surface and cartoon) representation of murine Tnks ARC2–3, bound to the murine Axin1 N‐terminus with two TBMs, shown in stick representation [protein data bank (PDB) code 3UTM] (Morrone *et al.,* 2012). In the crystal, ARC2–3 forms a dimer in which both copies of ARC2 are bound by one of the two TBMs of Axin1, respectively. (E) Detailed structural representation of the Axin1 TBMs (with indicated amino acid positions) on Tnks ARC2. The figure was generated by superimposing both ARC2–3 copies onto each other and displaying ARC2 bound to TBM1. Despite the N‐terminal insertion in TBM2, the arginine (typically at position 1) occupies the same sub‐pocket on the ARC, resulting in a looping out of the intervening residues. (F) Structural (transparent surface and cartoon) representation of a TNKS SAM polymer observed by X‐ray crystallography (PDB code 5JU5) (Mariotti *et al.,* 2016). (G) Avidity model for the interaction of AXIN and tankyrase, modified from Mariotti *et al*. (2016). Multivalency and polymerization of both tankyrase and AXIN enable avidity contributions in the interaction between both proteins. Note that tankyrase polymerization also promotes its PARP activity (Mariotti *et al.,* 2016; Riccio *et al.,* 2016).

TNKS and TNKS2 share a highly similar multi‐domain organization: five N‐terminal ankyrin repeat clusters (ARCs) for substrate binding are followed by a polymerizing sterile α motif (SAM) domain and a C‐terminal PARP catalytic domain (Figure 2A). With the exception of ARC3, the ARCs act as discrete substrate recognition domains for degenerate 8‐amino‐acid peptides of the consensus R‐[any]‐[any]‐[small hydrophobic or G]‐[D/E]‐G‐[no P]‐[D/E], termed the tankyrase‐binding motif (TBM) (Seimiya, 2002; Seimiya *et al.,* 2004; Guettler *et al.,* 2011). Briefly, an arginine at position 1 and glycine at position 6 of the TBM are essential. A small, hydrophobic residue or glycine is preferred at position 4, and acidic residues are optimal at positions 5 and 8, while proline is disallowed at position 7. Crystal structures of human TNKS2 ARC4 with various TBM peptides (Guettler *et al.,* 2011), of murine Tnks ARC2–3 in complex with Axin1 (Morrone *et al.,* 2012), of human TNKS ARC1–3 bound to TBM peptides derived from leucyl‐cystinyl aminopeptidase (LNPEP), combined with in‐solution structural studies (Eisemann *et al.,* 2016), and of human TNKS ARC5 bound to the TBM of USP25 (Xu *et al.,* 2017a), revealed the architecture of the ARCs and the principles of their substrate recognition. AXIN contains two TBMs in its N‐terminus (Morrone *et al.,* 2012), with the second motif bearing an unusual insertion (Figure 2B–E). The Tnks ARC2–3:Axin1 crystal structure shows a dimeric arrangement of ARC2–3 with each TBM peptide bound to one copy of ARC2 (Morrone *et al.,* 2012). AXIN is also able to contact two ARCs in the same Tnks molecule (Eisemann *et al.,* 2016). TNKS ARCs 1–3 adopt a relatively rigid asymmetric U‐shape, whereas ARCs 4–5 are more dynamic and flexibly linked to ARC1–3 (Eisemann *et al.,* 2016). Multiple AXIN binding sites in the ARCs and two TBMs in AXIN enable their cooperative interaction, but distance and conformational restraints create a preference for bivalent AXIN to either simultaneously bind ARCs 1 and 2, 4 and 5 or 2 and 5, with a preference for combinations involving ARC2, the strongest AXIN binder (Eisemann *et al.,* 2016). When binding to ARCs 2 and 5, AXIN induces a more compact conformation of the ARCs, which might place the PARP domain into closer proximity to ARC‐bound AXIN, in turn promoting AXIN PARylation (Eisemann *et al.,* 2016). Further studies are needed to explore this hypothesis. In the context of polymeric tankyrase, it appears equally likely that AXIN binds separate tankyrase molecules in the same tankyrase filament, with different implications for tankyrase conformation and potentially a further augmentation of cooperativity (Mariotti *et al.,* 2016; Riccio *et al.,* 2016).

Both TNKS and TNKS2 polymerize through their SAM domains (De Rycker and Price, 2004; Mariotti *et al.,* 2016; Riccio *et al.,* 2016). Recent crystallographic studies of the SAM domains revealed the primarily electrostatic nature of the head‐to‐tail SAM–SAM interfaces within the helical filament (Mariotti *et al.,* 2016; Riccio *et al.,* 2016) (Figure 2F), in agreement with a polymer model guided by NMR studies to identify the residues perturbed upon polymerization (DaRosa *et al.,* 2016). Compatible with the outward‐facing N‐ and C‐termini in the filament (DaRosa *et al.,* 2016; Mariotti *et al.,* 2016; Riccio *et al.,* 2016), full‐length tankyrase indeed polymerizes (De Rycker and Price, 2004; Mariotti *et al.,* 2016; Riccio *et al.,* 2016). TNKS and TNKS2 form cytoplasmic puncta rather than microscopically visible filaments, which may reflect the dynamic nature of the polymers (Mariotti *et al.,* 2016; Riccio *et al.,* 2016). This is consistent with observations made for other proteins containing polymerizing SAM domains (Isono *et al.,* 2013) and for polymerizing AXIN and Dishevelled (DVL)/DVL2 (Schwarz‐Romond *et al.,* 2007; Fiedler *et al.,* 2011; see Bienz, 2014). Supporting this view, polymerization‐deficient TNKS and TNKS2 mutants localize diffusely (Mariotti *et al.,* 2016; Riccio *et al.,* 2016). Luciferase reporter assays revealed that scaffolding through the ARCs and SAM domain is essential for tankyrase function in Wnt/β‐catenin signalling (Mariotti *et al.,* 2016; Riccio *et al.,* 2016). Surprisingly, tankyrase can substantially drive Wnt/β‐catenin activity even in the absence of its catalytic PARP activity, entirely through scaffolding (Huang *et al.*, 2009; Mariotti *et al.,* 2016). Tankyrase polymerization enables productive interactions with the limited pool of AXIN, through avidity effects arising from multivalency and polymerization in both tankyrase and AXIN (Fiedler *et al.,* 2011; Mariotti *et al.,* 2016) (Figure 2G), a requirement that appears overridden by AXIN overexpression (Riccio *et al.,* 2016, and our unpublished observations). The SAM domain and SAM domain‐dependent polymerization are also required for full tankyrase PARP activity (De Rycker and Price, 2004; Levaot *et al.,* 2011; Mariotti *et al.,* 2016; Riccio *et al.,* 2016). Interestingly, while PARP activity is dispensable for tankyrase‐driven Wnt/β‐catenin signalling under basal conditions (Mariotti *et al.,* 2016), it is necessary for tankyrase to potentiate Wnt‐induced β‐catenin activity (Riccio *et al.,* 2016). This may well reflect the recently discovered requirement of AXIN PARylation in the formation of Wnt‐induced signalosomes (see below) (Yang *et al.,* 2016; Wang *et al.,* 2016a) (Figure 1B).

## A potential role of tankyrase in the formation of β‐catenin degradasomes

**Figure 3:**
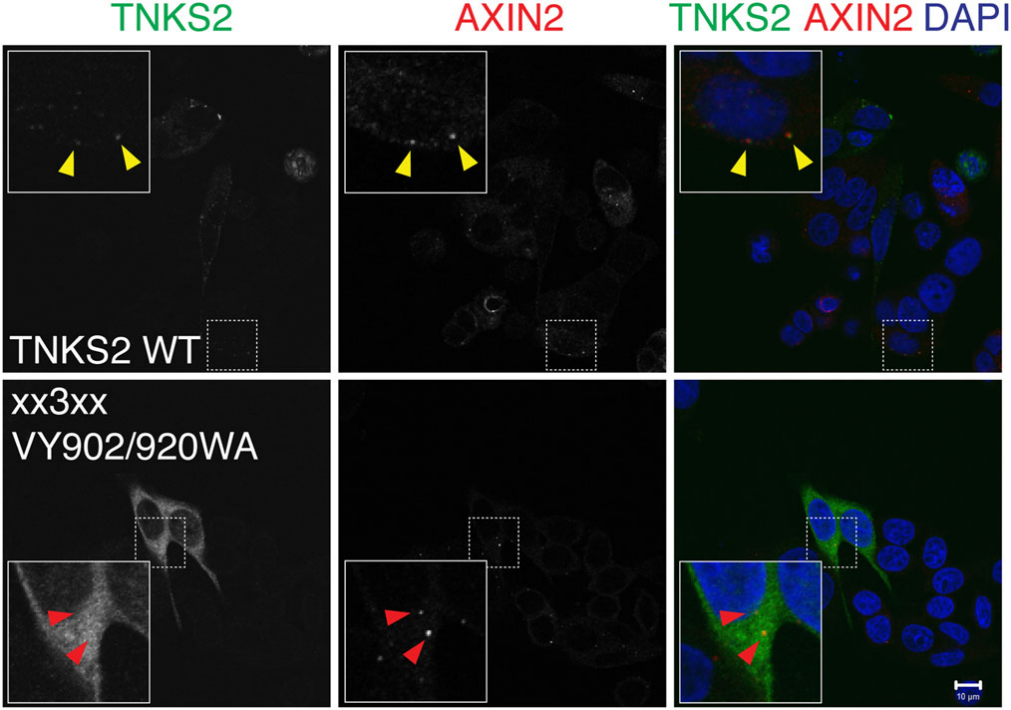
β‐catenin degradasomes induced by TNKSi. SW480 CRC cells were treated with the TNKSi XAV939 and immunostained for AXIN2 (red) and transiently expressed epitope‐tagged wild‐type TNKS2 (TNKS2 WT) or a TNKS2 mutant variant deficient in substrate binding (through site‐directed mutation of ARCs 1, 2, 4 and 5) and polymerization (TNKS2 xx3xx VY903/920WA) (green). Yellow arrowheads indicate colocalization of TNKS2 and AXIN2 in β‐catenin degradasomes; red arrowheads indicate absence of colocalization for the scaffolding‐defective mutant variant of TNKS2. The figure was modified from Mariotti *et al*. (2016).

When AXIN is overexpressed or stabilized in APC‐mutant cells by tankyrase inhibition (using the inhibitors JW67, JW74, JW55, XAV939 or G007‐LK; see Table 1 and below for a discussion of inhibitors), it accumulates in cytoplasmic puncta, together with other β‐catenin destruction complex components, including GSK3β, APC, β‐catenin, β‐transducin repeats‐containing protein (β‐TRCP) and tankyrase (Waaler *et al.,* 2011, 2012; de la Roche *et al.,* 2014; Thorvaldsen *et al.,* 2015; Martino‐Echarri *et al.,* 2016) (Figure 3). The puncta gradually disappear upon removal of the tankyrase inhibitor (TNKSi) and re‐establish with subsequent inhibitor treatment (Thorvaldsen *et al.,* 2015). The puncta, referred to as β‐catenin degradasomes, are understood to be ‘morphological correlates’ of β‐catenin destruction complexes, which without tankyrase inhibition are not visible by light microscopy due to the normally low AXIN levels (de la Roche *et al.,* 2014; Thorvaldsen *et al.,* 2015; Pedersen *et al.,* 2016). Several features illustrate functionality of these degradasomes. Firstly, they contain phosphorylated β‐catenin and also colocalize with ubiquitin and β‐TRCP, a component of the E3 ubiquitin ligase responsible for β‐catenin ubiquitylation (Thorvaldsen *et al.,* 2015). Secondly, live‐cell imaging by fluorescence recovery after photobleaching (FRAP) in SW480 cells showed that β‐catenin is rapidly turned over in degradasomes (Thorvaldsen *et al.,* 2015), demonstrating their capacity to degrade β‐catenin.

**Table 1:** Tankyrase inhibitors

No.	Tankyrase inhibitor	TNKS IC_50_ (nM)	TNKS2 IC_50_ (nM)	PARP1 [PARP2] IC_50_ (nM)	IC_50_ in Wnt reporter assay (nM)	Binding site	References
1	(1) 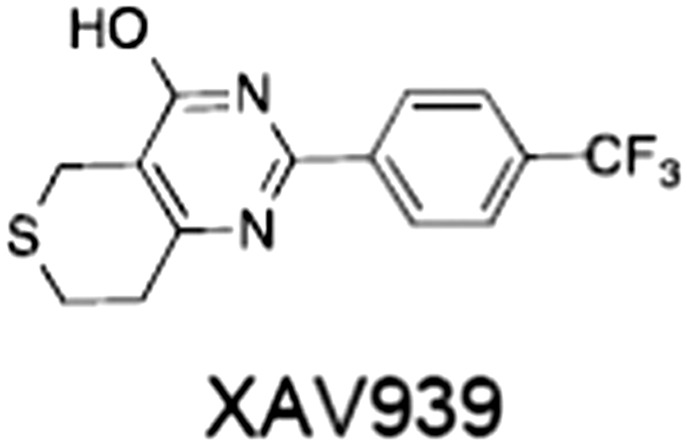	11	4	PARP domain: 2194 [114] full‐length:74 [27]	78 (HEK293, Wnt‐3a)	Nicotinamide	(Huang *et al.,* 2009; Karlberg *et al.,* 2010; Kirby *et al.,* 2012; Shultz *et al.,* 2012; Thorsell *et al.,* 2017)
2	(2) 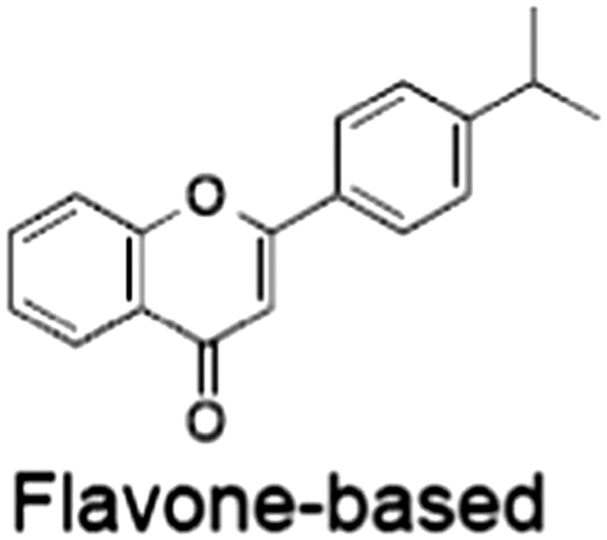	6	72	19 100 [34900]	‐	Nicotinamide	(Narwal *et al.,* 2013a, 2013b)
3	(3) 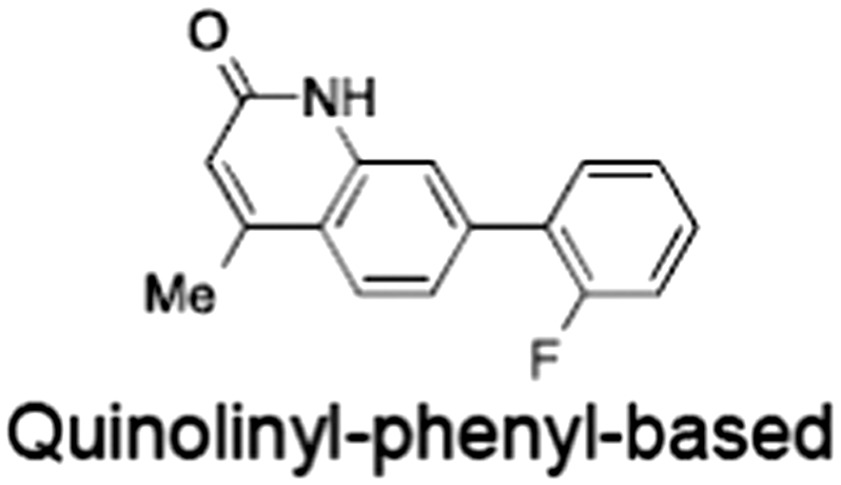	860	52	>10 000 [>10 000]	‐	Nicotinamide	(Larsson *et al.,* 2013)
4	(4) 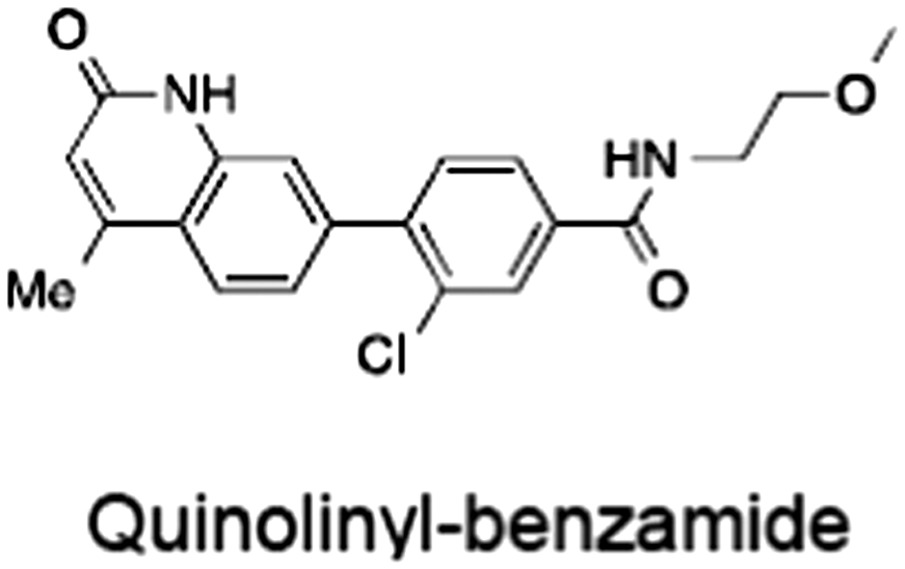	‐	9	‐	‐	Nicotinamide	(Larsson *et al.,* 2013)
5	(5) 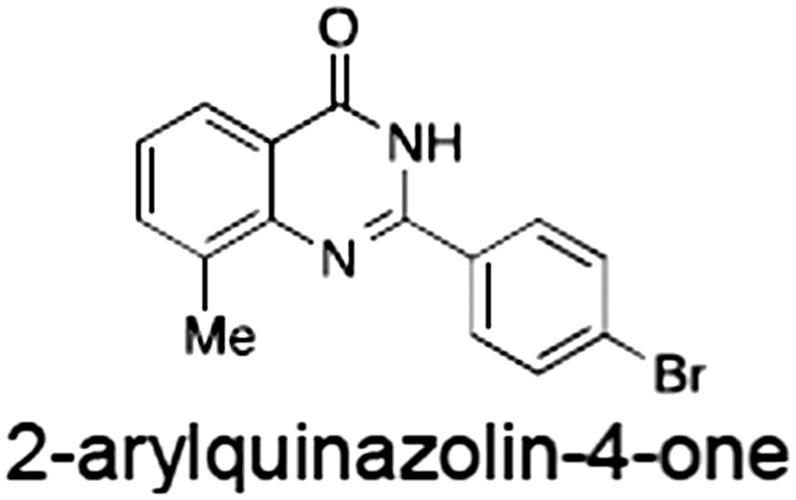	32	9	>5000	‐	Nicotinamide	(Nathubhai *et al.,* 2013, 2016)
6	(6) 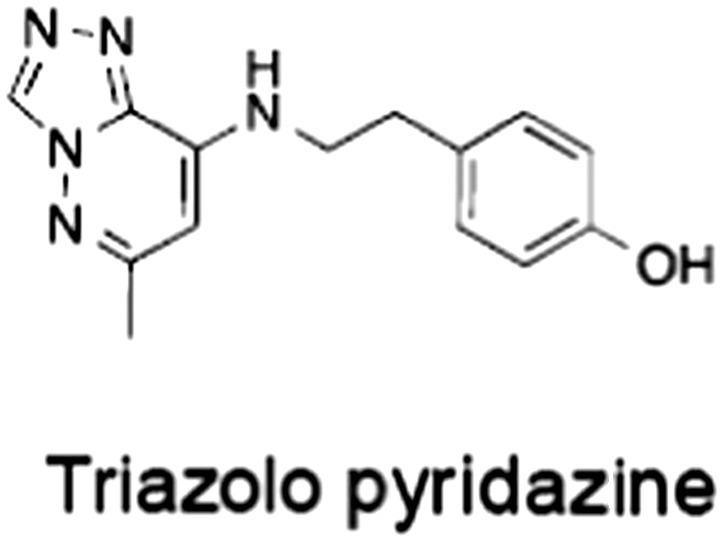	12	200	>10 000 [>10 000]	‐	Nicotinamide	(Liscio *et al.,* 2014)
7	(7) 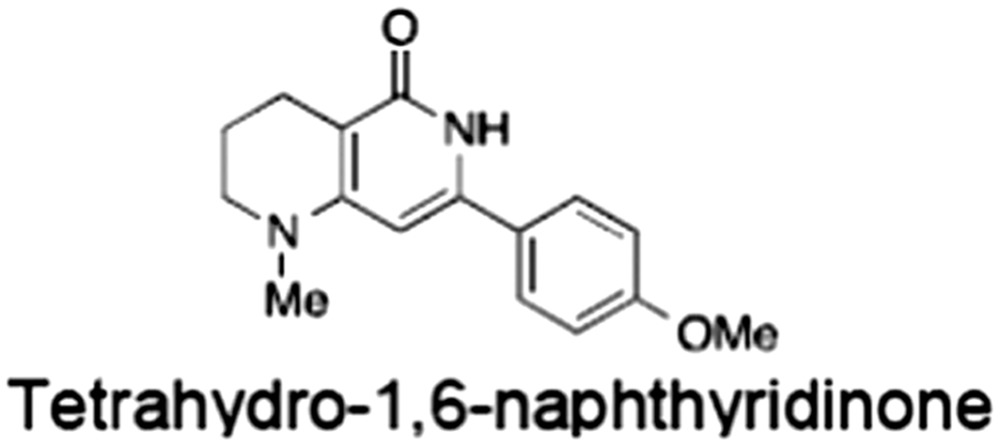	1.7	1.1	3400	‐	Nicotinamide	(Kumpan *et al.,* 2015)
8	(8) 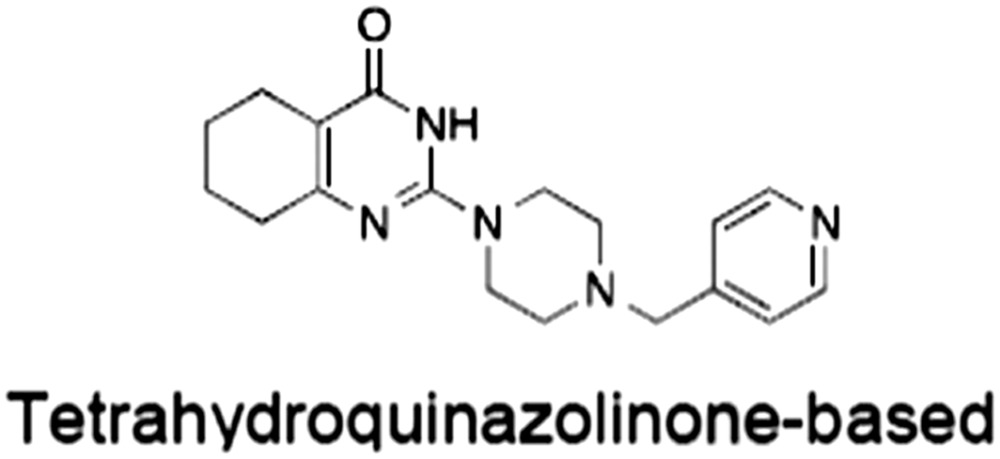	370	71	5900 [4200]	‐	Nicotinamide	(Nkizinkiko *et al.,* 2015)
9	(9) 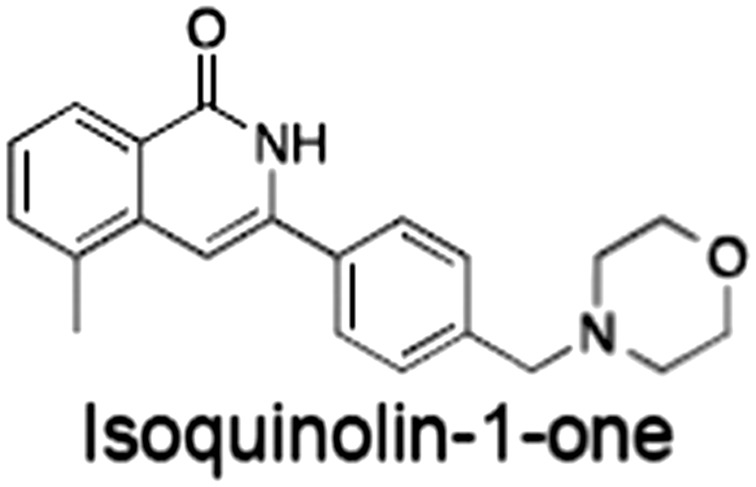	12	‐	‐a	25 (DLD‐1)	Nicotinamide	(Elliott *et al.,* 2015; Paine *et al.,* 2015)
10	(10) 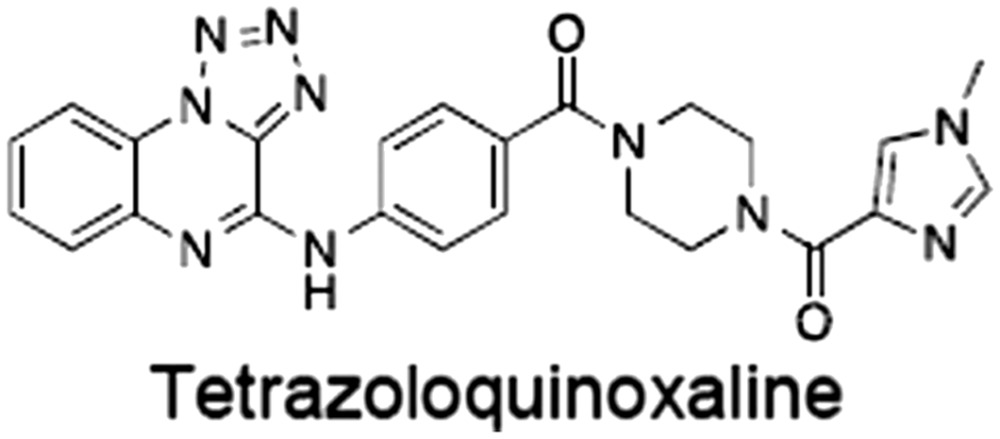	32	‐	>1995	32 (HEK293, Wnt‐3a)	Nicotinamide	(Thomson *et al.,* 2017)
11	(11) 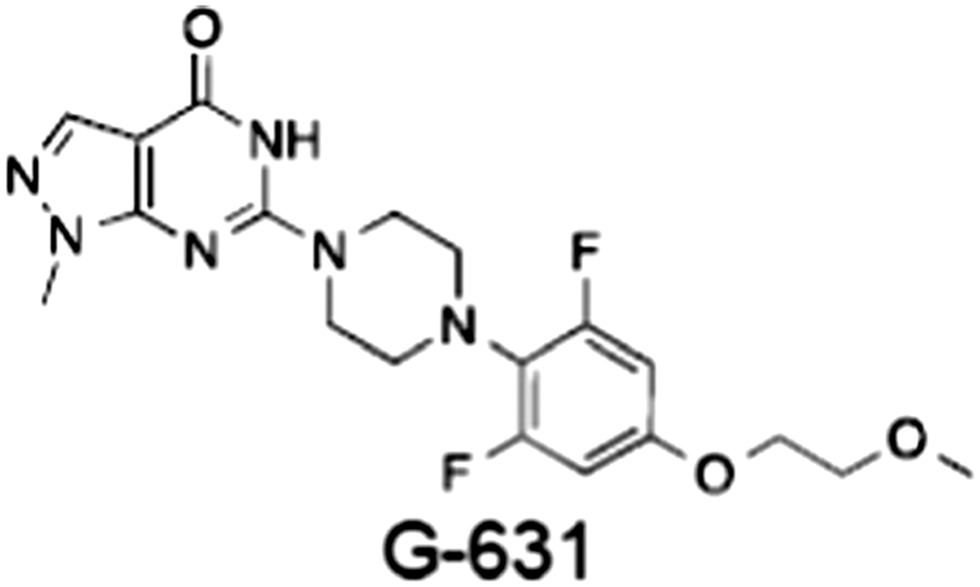	8	24	>10 000	8 (HEK293, Wnt‐3a)	Nicotinamide	(patent by Feng *et al.,* 2013; Zhong *et al.,* 2016b)
12	(12) 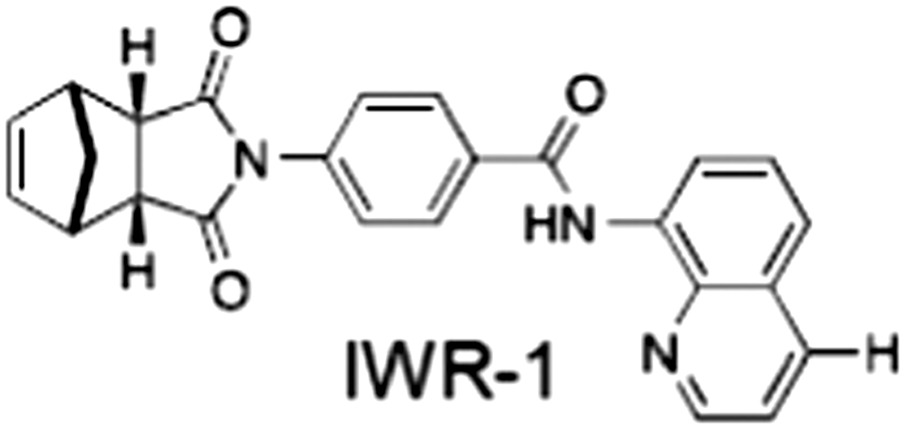	131	56	>18 750 [>18 750]	180 (L cells, Wnt‐3a)	Adenosine	(Chen *et al.,* 2009; Huang *et al.,* 2009; Lu *et al.,* 2009; Gunaydin *et al.,* 2012)
13	(13) 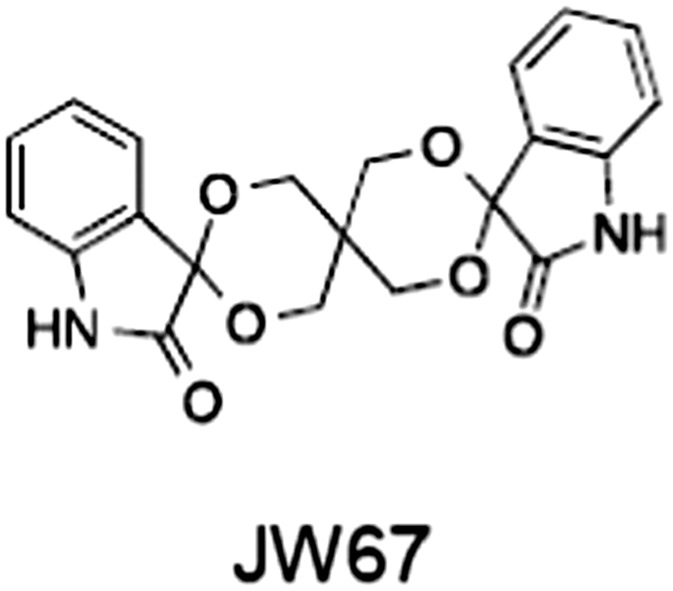	‐	‐	‐	1170 (HEK293, Wnt‐3a)	Unknown	(Waaler *et al.,* 2011)
14	(14) 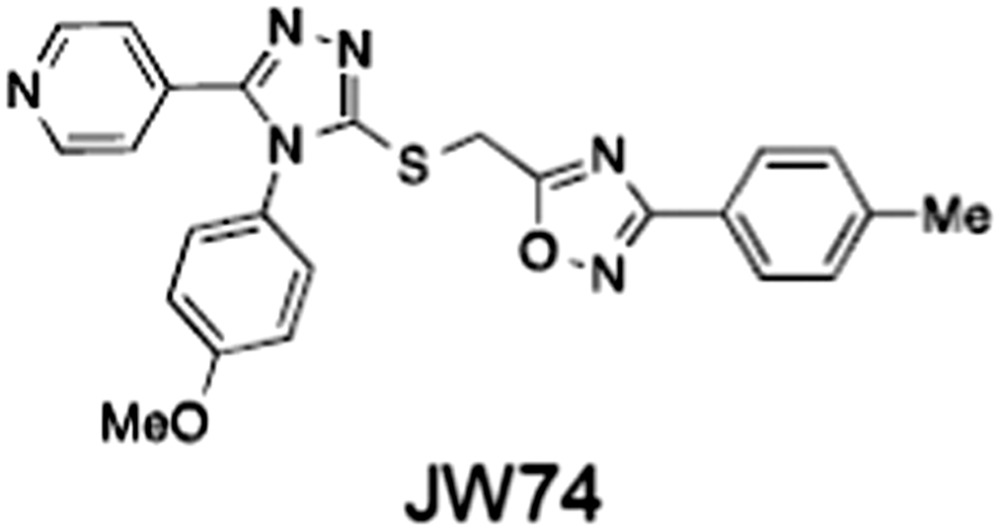	2550	650	‐	790 (HEK293, Wnt‐3a)	Adenosine	(Waaler *et al.,* 2011; Shultz *et al.,* 2012; Voronkov *et al.,* 2013)
15	(15) 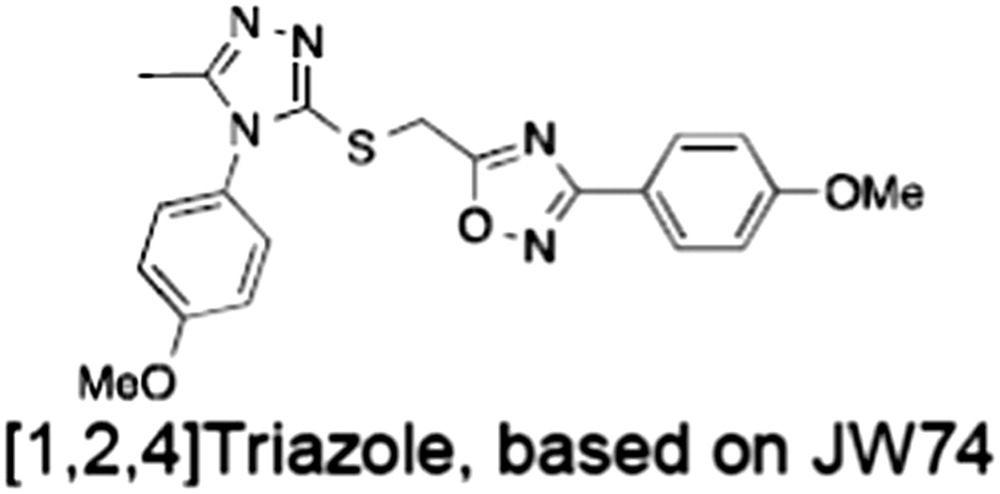	‐	33	>19 000 [>19 000]	215 (HEK293, Wnt‐3a)	Adenosine	(Shultz *et al.,* 2012)
16	(16) 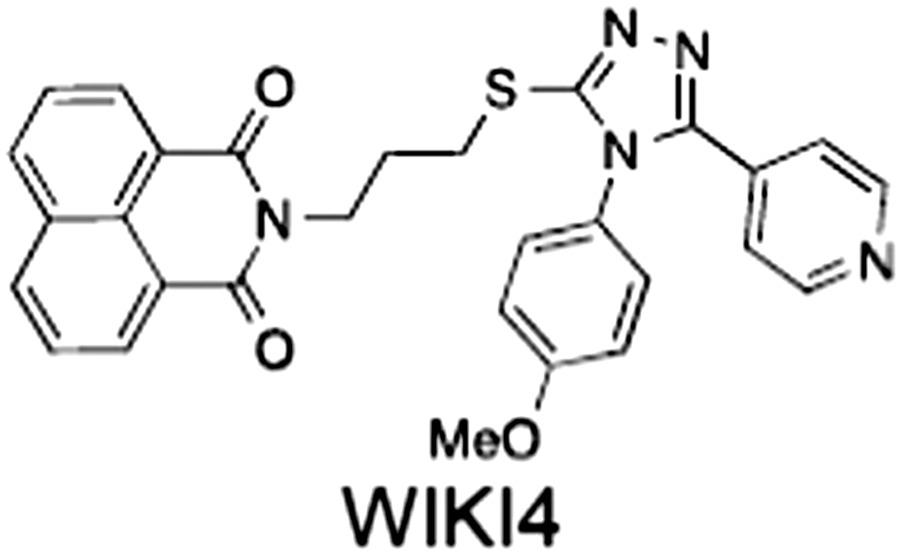	26	15	>10 000 [>10 000]	‐	Adenosine	(James *et al.,* 2012; Haikarainen *et al.,* 2013)
17	(17) 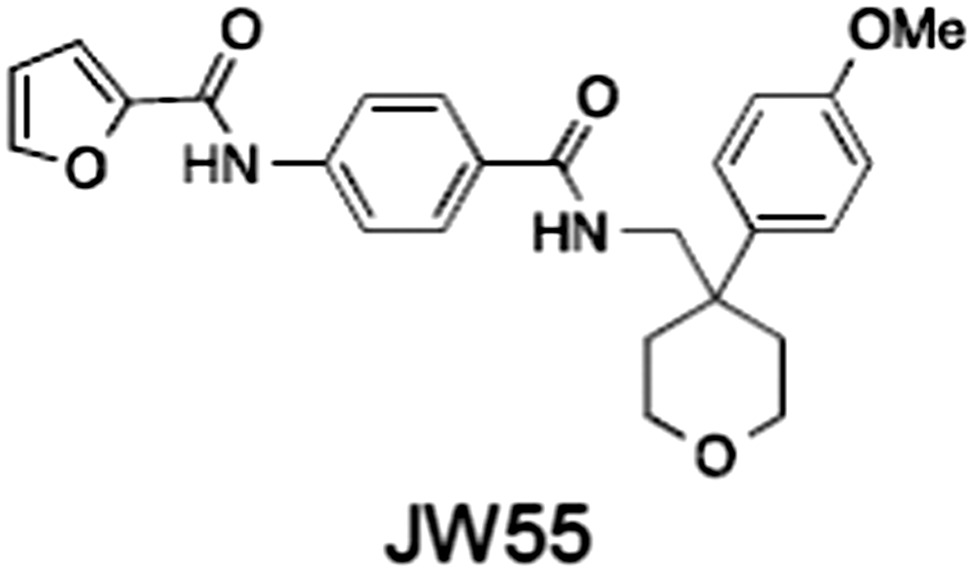	1800	2010	‐	1230 (HEK293, Wnt‐3a)	Adenosine	(Waaler *et al.,* 2012; Haikarainen *et al.,* 2016)
18	(18) 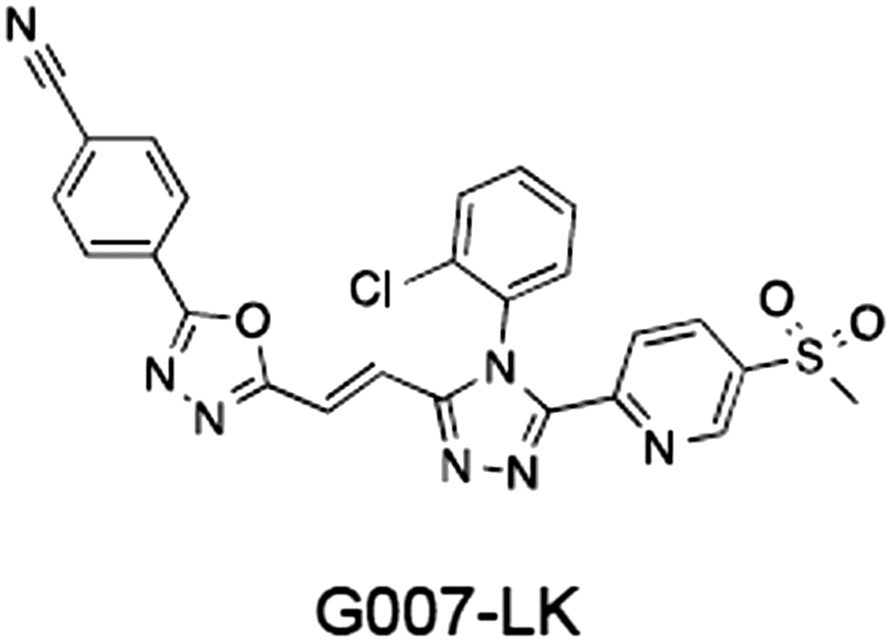	46	25	>10 000 [>10 000]	50 (HEK293, Wnt‐3a)	Adenosine	(Lau *et al.,* 2013; Voronkov *et al.,* 2013)
19	(19) 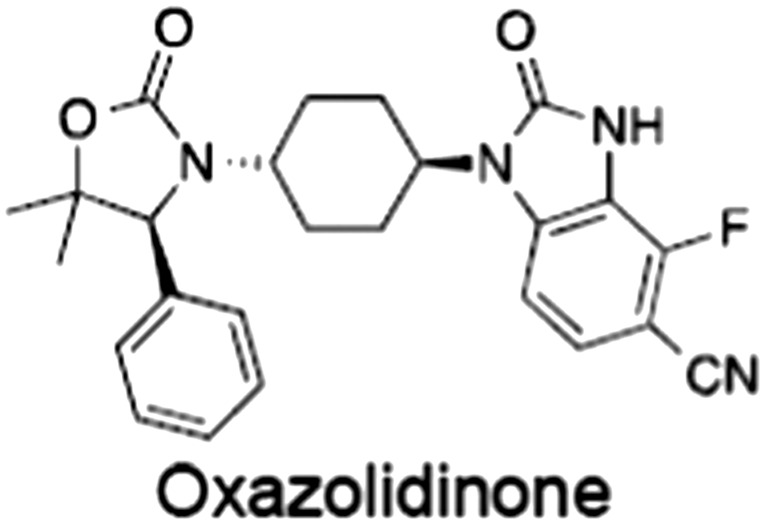	1	‐	>85 000 [>170 000]	‐	Adenosine	(Bregman *et al.,* 2013a)
20	(20) 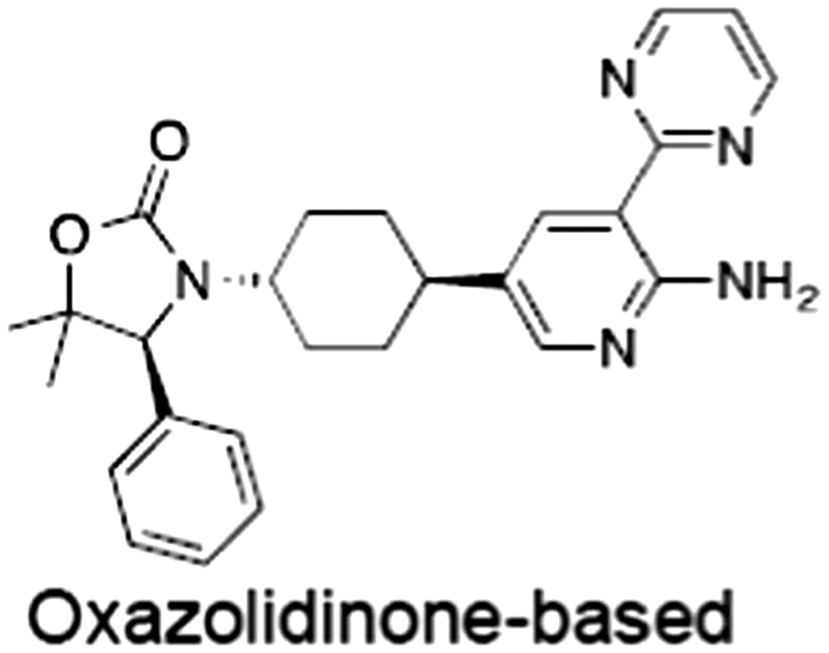	2	2	>85 000 [>170 000]	12 (DLD‐1)	Adenosine	(Huang *et al.,* 2013)
21	(21) 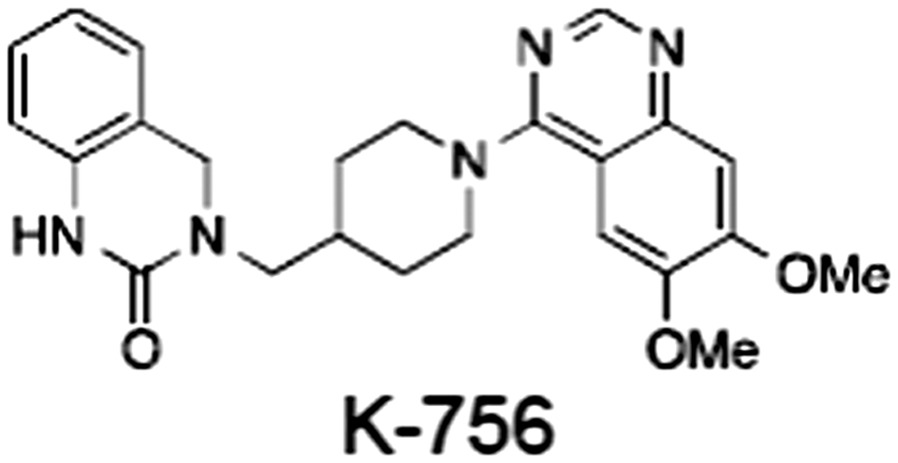	31	36	‐	110 (DLD‐1)	Adenosine	(Okada‐Iwasaki *et al.,* 2016)
22	(22) 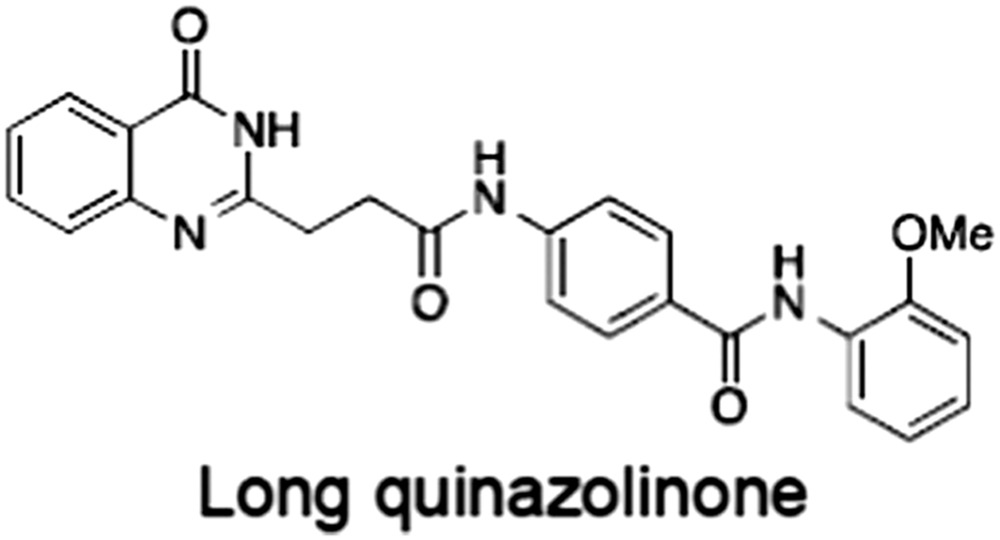	8	2	[931]	36 000 (HEK293, Wnt‐3a)	Dual site	(Bregman *et al.,* 2013b)
23	(23) 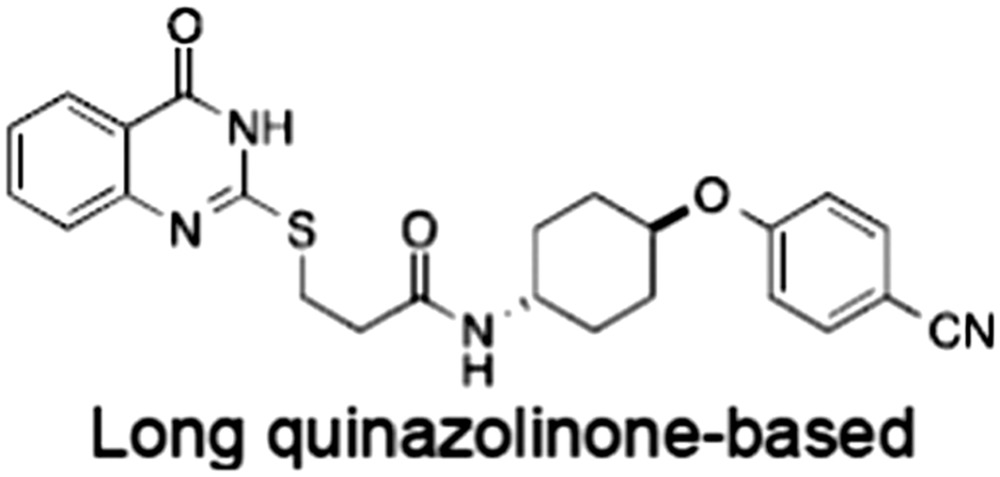	0.2	2.5	‐	1.3 (DLD‐1)	Dual site	(Hua *et al.,* 2013)
24	(24) 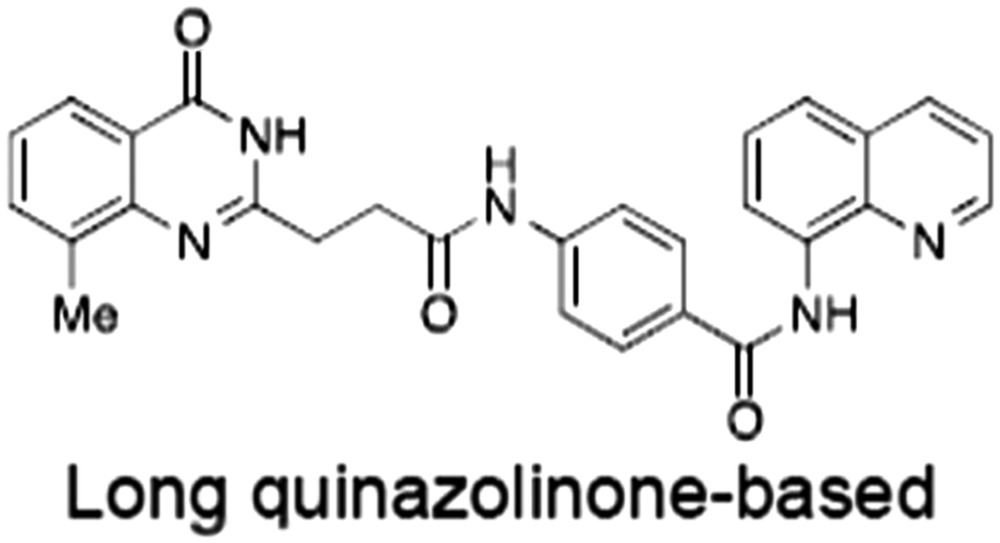	5.1	0.1	6500 [11600]	37 (HEK293, Wnt‐3a)	Dual site	(Nathubhai *et al.,* 2017)
25	(25) 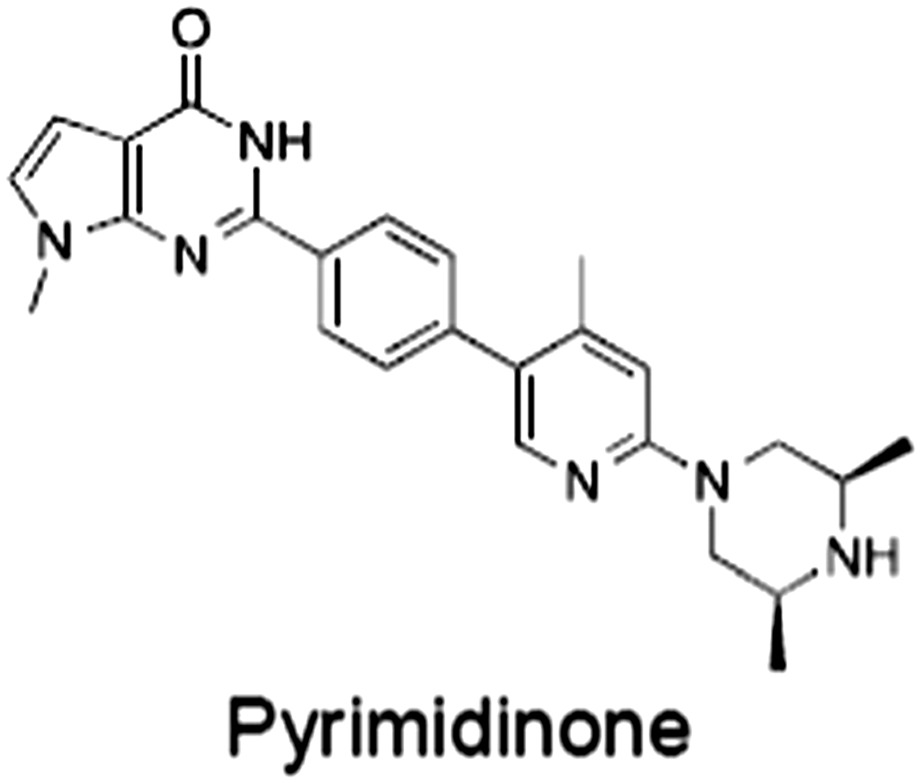	3	1	2.0 [0.5]	5 (DLD‐1)	Dual site	(Johannes *et al.,* 2015a)
26	(26) 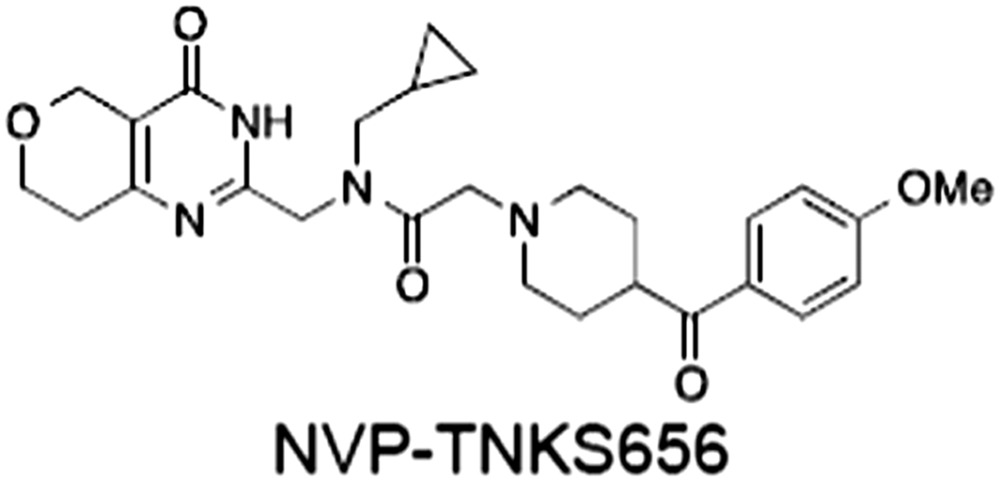	‐	6	>19 000 [>30 000]	3.5 (HEK293, Wnt‐3a)	Dual site	(Shultz *et al.,* 2013)

The structures and properties of reported tankyrase‐selective inhibitors are shown, grouped according to binding site. Dual site binders occupy both the nicotinamide and adenosine subsites. One typical structure is given if numerous similar compounds in a series were detailed in the literature. Biochemical IC_50_ values (i.e. corresponding to inhibition of PARP activity) for TNKS, TNKS2, PARP1 and PARP2 and IC_50_ values in Wnt/β‐catenin reporter assays are listed if reported. ‘‐’ indicates that values have not been reported. Note that NVP‐TNKS656 is reported as a ‘triple site binder’, also interacting with a hydrophobic ‘nook’ adjacent to the phosphate‐binding groove (Shultz *et al.,* 2012, 2013).

a Although the IC_50_ for PARP1/2 was not determined for compound 9 (Elliott *et al.,* 2015), other compounds in the same series displayed IC_50_ values for PARP1/2 of at least one order of magnitude above those for TNKS (Elliott *et al.,* 2015; Paine *et al.,* 2015).

Two recent studies suggest that tankyrase plays a structural role in degradasome formation (Thorvaldsen *et al.,* 2015; Martino‐Echarri *et al.,* 2016). Correlative light and electron microscopy suggests that TNKS‐GFP‐containing degradasomes in TNKSi‐treated SW480 cells correspond to electron‐dense and possibly filamentous substructures (Thorvaldsen *et al.,* 2015), perhaps reflecting the polymeric nature of both AXIN and tankyrase. Simultaneous silencing of both tankyrases abolishes degradasome formation (Martino‐Echarri *et al.,* 2016). Other studies have shown that, like tankyrase inhibition, TNKS/TNKS2 RNAi increases the levels of AXIN1/2 (Huang *et al.,* 2009). Strikingly, despite increased AXIN levels, degradasomes are absent under TNKS/TNKS2‐depleted conditions (Martino‐Echarri *et al.,* 2016), supporting a direct, structural role for tankyrase in degradasome formation. FRAP studies have shown that TNKS stably resides in degradasomes, similarly to AXIN (Schwarz‐Romond *et al.,* 2005; Thorvaldsen *et al.,* 2015), although tankyrase and AXIN dynamics have not yet been studied in the same cell. The multivalent interactions of AXIN with tankyrase and the avidity‐enhancing polymerization of both proteins may underlie a scaffolding function of tankyrase in degradasome formation (Mariotti *et al.,* 2016) (Figure 2G). Tankyrase polymerization may be promoted by its catalytic inhibition (De Rycker and Price, 2004), which might offer a potential explanation for the TNKSi‐induced stabilization of degradasomes. APC2, which was recently reported to bind tankyrase (Croy *et al.,* 2016), may also contribute to the avidity‐dependent degradasome assembly, given its numerous AXIN and β‐catenin binding sites.

AXIN2 protein levels rapidly increase upon tankyrase inhibition in SW480 cells, whereas AXIN1 levels do not change until much later (Pedersen *et al.,* 2016; Thorvaldsen *et al.,* 2017). Knockdown of AXIN2 but not AXIN1 prevents degradasome formation (Thorvaldsen *et al.,* 2017), indicating that AXIN2 is the predominant AXIN scaffold in these cells. The TNKSi‐induced accumulation of AXIN2 is dependent on new protein synthesis (Thorvaldsen *et al.,* 2017) and active transcription of the *AXIN2* gene (Pedersen *et al.,* 2016). Proteasome inhibition leads to increased levels of phosphorylated β‐catenin in degradasomes (Thorvaldsen *et al.,* 2015), but prolonged proteasome inhibition impairs degradasome formation (Martino‐Echarri *et al.,* 2016; Pedersen *et al.,* 2016). Pedersen *et al*. (2016) proposed that the transcription factor Forkhead box protein M1 (FoxM1), whose activating phosphorylation is suppressed by proteasome inhibition, controls the *AXIN2* gene. *AXIN2* is also a Wnt/β‐catenin target gene as part of a negative feedback loop and is highly expressed in APC‐mutated CRC cells (Yan *et al.,* 2001; Jho *et al.,* 2002; Lustig *et al.,* 2002). It is not known why TNKSi induces β‐catenin degradasomes in many CRC cells but far less so in cells with an intact Wnt/β‐catenin pathway (de la Roche *et al.,* 2014). It is possible that tankyrase inhibition strongly represses the *AXIN2* gene in Wnt/β‐catenin wild‐type cells. Conversely, APC‐mutant cells might still display residual *AXIN2* transcription with AXIN2 protein accumulation arising from continued AXIN2 synthesis and blocked PARdU, leading to the formation of large degradasomes. Degradasome assembly depends on the concentrations of their components (Bienz, 2014), and it is likely that fully functional degradasomes also form in Wnt/β‐catenin wild‐type cells, but these structures remain small.

## The structural basis of PARdU

**Figure 4:**
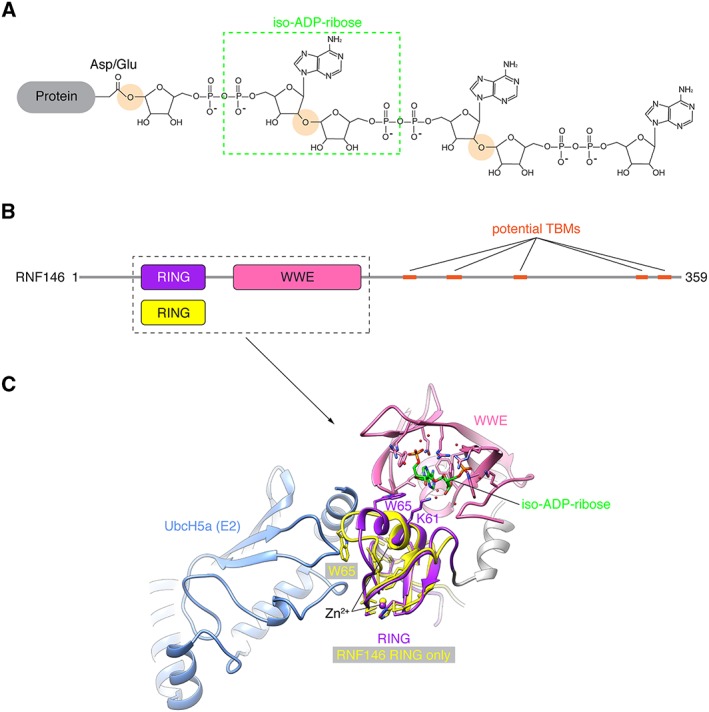
Allosteric regulation of RNF146/Iduna by PAR binding. (A) Structure of a linear PAR chain, here attached to Asp/Glu. The O‐glycosidic bonds linking ADP‐ribose units are highlighted. The green box indicates iso‐ADP‐ribose. Tankyrase is thought to generate linear PAR chains (Rippmann *et al.,* 2002); PAR branches are therefore omitted. (B) Domain organization of human RNF146. Potential TBMs are indicated (DaRosa *et al.,* 2015). The boxed area, which includes the isolated RING domain, corresponds to (C). (C) Structural representation of RNF146 bound to iso‐ADP‐ribose [protein data bank (PDB) code 4QPL] and the E2 enzyme UbcH5a (DaRosa *et al.,* 2015). The isolated RING domain of RNF146 (PDB code 2D8T, one representative of the solution structure ensemble) is superimposed (DaRosa *et al.,* 2015). Domains and corresponding Zn^2+^ ions are colour‐coded as in (B). Key residues involved in PAR coordination and the allosteric switch are shown in stick representation. Note the clash occurring between the RING domain (yellow) and the E2 enzyme (blue) in the absence of the PAR ligand, and the conformational change upon PAR binding, resulting in a reorientation of Trp65.

Once PARylated, AXIN is engaged by the PAR‐binding E3 ubiquitin ligase RNF146/Iduna (Callow *et al.,* 2011; Kang *et al.,* 2011; Zhang *et al.,* 2011). RNF146 consists of an RING domain followed by a PAR‐binding WWE domain and an extended C‐terminus, which is predicted to be largely unstructured (Figure 4B). The PAR‐dependency of the enzyme suggested an allosteric activation mechanism; in addition, PAR may serve as a scaffold to enable increased local concentrations of the enzyme (Callow *et al.,* 2011; Kang *et al.,* 2011; Zhang *et al.,* 2011). A crystal structure of RNF146 (RING‐WWE) bound to iso‐ADP‐ribose, an internal unit of PAR (Figure 4A), and an E2 conjugating enzyme is compatible with the allosteric activation of RNF146 (DaRosa *et al.,* 2015) (Figure 4C). Iso‐ADP‐ribose not only binds the WWE but also the RING domain (Wang *et al.,* 2012; DaRosa *et al.,* 2015) and appears to induce restructuring of a loop, which in the apo form of the RING domain extends into the E2‐E3 enzyme contact region, thereby precluding the interaction (Figure 4C). The restructured loop residues become part of an extended central helix in the RING domain, which no longer obstructs E2 binding (Figure 4C), a model supported by NMR spectroscopy and mutagenesis (DaRosa *et al.,* 2015). The extended C‐terminus of RNF146 directly binds tankyrase *via* five proposed TBMs (DaRosa *et al.,* 2015) (Figure 4B). It is noteworthy that all of these TBMs are atypical in their length or sequence, suggesting that they might be of relatively low individual affinity and may need to act collectively to recruit tankyrase. A model in which the tankyrase:RNF146 complex can still bind other TBM‐containing proteins *via* the multivalent ARCs implies that the substrate specificity of tankyrase determines RNF146 substrate specificity (DaRosa *et al.,* 2015). Like many PARPs, tankyrase modifies itself, and not all tankyrase binders are also PARylated (Bae, 2002; Guettler *et al.,* 2011; Bisht *et al.,* 2012). This raises the interesting possibility that non‐PARylated tankyrase binders may still be ubiquitylated by RNF146 present in the complex, with RNF146 getting activated by tankyrase auto‐PARylation or PAR attachment to different, simultaneously bound substrates.

While tankyrase RNAi stabilizes AXIN and reduces β‐catenin‐dependent transcription in certain CRC cell lines (Huang *et al.,* 2009; Callow *et al.,* 2011), silencing of RNF146 fails to increase AXIN levels in HCT‐15 or SW480 CRC cells, both of which bear APC truncations, and does not inhibit Wnt/β‐catenin signalling in HCT‐15 cells (Callow *et al.,* 2011). This suggests that there are alternative pathways for the degradation of PARylated AXIN in these cells (Callow *et al.,* 2011). The existence of numerous RING‐type E3 ubiquitin ligases with PAR‐binding WWE domains suggests that functional redundancies may exist (Wang *et al.,* 2012).

## Tankyrase and APC set a threshold for Wnt responsiveness by limiting AXIN abundance

To assess the maximum AXIN level still allowing productive Wnt/β‐catenin signalling, Wang *et al*. (2016b) engineered flies overexpressing C‐terminally V5 epitope‐tagged Axin (Axin‐V5). (Note that *Drosophila* has a single Axin paralogue; see Figure 2C.) Despite an up to fourfold Axin‐V5 overexpression, flies develop normally with only a mild defect attributable to inhibited Wg signalling (Wang *et al.,* 2016b), in agreement with previous studies (Peterson‐Nedry *et al.,* 2008). This suggested that Axin‐V5 is still subject to physiological regulation at this level. Likewise, loss of tankyrase (of which there is also only a single paralogue in *Drosophila*) results in a mild (two‐ to threefold) increase in endogenous Axin abundance in larvae, without measurable developmental effects (Feng *et al.,* 2014; Wang *et al.,* 2016b, 2016c). However, developmental defects and loss of Wg/Armadillo target gene expression are observed when Axin‐V5 is expressed in wing imaginal discs in a tankyrase null background, which results in a further threefold increase of Axin‐V5 levels, positioning the inhibitory threshold for Axin three‐ to ninefold above endogenous‐regulated levels (Wang *et al.,* 2016b). This illustrates tankyrase's strong capacity to buffer negative regulation of Wg signalling by Axin, a phenomenon also seen in mammalian cells (Mariotti *et al.,* 2016). These observations are compatible with the previous finding that knockdown of tankyrase in the developing wing only leads to a Wg phenotype if Axin is simultaneously overexpressed (Feng *et al.,* 2014).

Besides their role in promoting β‐catenin/Armadillo degradation, Apc/Apc2 also play a positive role in regulating Wg/Armadillo signalling by post‐transcriptionally limiting Axin levels (Takacs *et al.,* 2008; Wang *et al.,* 2016b). Accordingly, Axin‐V5 accumulates in imaginal discs lacking Apc1 and Apc2 (Wang *et al.,* 2016b). The regulation of Axin levels by Apc is strictly dependent on the Apc‐binding regulator of G‐protein signalling (RGS) domain of Axin, suggesting that a physical interaction between the two proteins is required (Wang *et al.,* 2016b). Surprisingly, while the ability of tankyrase to destabilize Axin is independent of Apc, the TBM of Axin is necessary for Axin destabilization by Apc, suggesting that tankyrase binding is required (Wang *et al.,* 2016b). Alternatively, the Axin N‐terminus may play a tankyrase‐independent role in Axin regulation by Apc. The mechanism by which Apc destabilizes Axin remains unclear, and it will be interesting to decipher whether PARdU is involved. A recent report suggests the existence of a TBM in Apc2 (Croy *et al.,* 2016). Tankyrase may bind to both Apc2 and Axin, thereby providing an additional scaffolding role. The existence of partially separable degradation pathways for Axin, through tankyrase and Apc/Apc2, may explain the mild Tnks null phenotype in flies (Wang *et al.,* 2016b). By analogy, Wang *et al*. (2016b) propose that if AXIN regulation by APC is lost in APC‐mutant CRC cells, then these cells might be particularly susceptible to tankyrase inhibition, in contrast to APC wild‐type cells, opening the possibility for selectively targeting APC‐mutant cells. This is an interesting idea for further exploration.

## Tankyrase mouse models

The physiological role of tankyrase in Wnt/β‐catenin signalling is still far from being fully understood. In mice, loss of both tankyrases gives rise to embryonic lethality, without indication that lethality is attributable to defective Wnt/β‐catenin signalling (Chiang *et al.,* 2008). Individual knockout of either Tnks or Tnks2 results in non‐pathogenic phenotypes, but again, there is no sign of a dysregulated Wnt pathway at the phenotypic level (Chiang *et al.,* 2006; Hsiao *et al.,* 2006; Chiang *et al.,* 2008). One may speculate that embryonic lethality in the double knockout arises from another essential role of tankyrase in development or pleiotropy due to the complex involvement of tankyrase in many biological processes, masking a function in Wnt/β‐catenin signalling. Moreover, these observations point toward a substantial functional redundancy between the two tankyrases. In the absence of conditional Tnks/Tnks2 knockout mice, the role of tankyrase in Wnt‐dependent physiology is difficult to assess. However, an Axin2 V26D mutation, which maps to the stronger N‐terminal TBM of murine Axin2 (Figure 2C), also results in embryonic lethality but with an identifiable Wnt phenotype (Qian *et al.,* 2011). The mutation is expected to abolish tankyrase binding (Guettler *et al.,* 2011), compatible with increased Axin2 levels and reduced Wnt/β‐catenin signalling in most tissues (Qian *et al.,* 2011). Surprisingly, increased rather than decreased Wnt/β‐catenin signalling is seen in the late primitive streak, a structure that marks the beginning of gastrulation and the definition of body axes, and consequential formation of ectopic tails (Qian *et al.,* 2011). This observation points to complex functions of the tankyrase‐Axin interaction. The Axin2 mutation likely exposes a physiological role of tankyrase in Wnt/β‐catenin signalling that may have been masked in the Tnks/Tnks2 double‐knockout mice, although alternative functions of the Axin N‐terminus cannot be excluded. A study otherwise focussing on the role of tankyrase in glucose metabolism corroborates the tankyrase‐Axin‐β‐catenin link *in vivo*: adipocyte‐specific loss of the Tnks catalytic domain stabilizes Axin1 and reduces the levels of active β‐catenin in adipose tissue (Zhong *et al*., 2016a).

## Tankyrase controls adult intestinal stem cell homeostasis in *Drosophila*

A more detailed analysis of Tnks mutant flies revealed a sharp drop in viability upon nutrient limitation, paralleled by an accumulation of Axin and hyperproliferation of intestinal stem cells (ISCs) in the midgut (Wang *et al.,* 2016c). The *Drosophila* midgut can be subdivided into several morphologically and physiologically distinct domains. It displays high levels of Wg target gene transcription close to the inter‐domain boundaries, which may represent source areas for the Wg ligand (Buchon *et al.,* 2013; Tian *et al.,* 2016). Loss of tankyrase distant from the midgut‐hindgut boundary, where β‐catenin/Armadillo activity is low, abolishes the activation of Wg reporters in this region (Wang *et al.,* 2016c). This is not the case in areas close to the midgut‐hindgut boundary, where Wg signalling is high. The authors suggested that by counteracting Axin, tankyrase amplifies β‐catenin/Armadillo activity in compartments of otherwise low pathway activity. They hypothesized that context‐specific roles of tankyrase may explain seemingly contradicting *in vivo* functions of tankyrase in flies and zebrafish (Huang *et al.,* 2009; Feng *et al.,* 2014) and that similar mechanisms may be in place where Wnt gradients are observed in vertebrates, for example, in the gut (Wang *et al.,* 2016c). (See below for a further discussion of tankyrase's roles in the gastrointestinal tract.) Interestingly, hyperproliferation of ISCs is non‐cell‐autonomous: it does not result from tankyrase loss or inhibited Wg/Armadillo signalling in ISCs but in enterocytes (Tian *et al.,* 2016; Wang *et al.,* 2016c). The authors suggested that decreased Wg signalling in enterocytes upon loss of tankyrase promotes JAK/STAT signalling in ISCs, which in turn drives their proliferation during homeostasis.

## A role of AXIN PARylation in Wnt signalosome assembly

While tankyrase's role in PARdU of AXIN is well established, two recent studies point to a novel function in Wnt signalosome assembly (Yang *et al.,* 2016; Wang *et al.,* 2016a) (Figure 1B). During *Drosophila* embryonic development, the abundance of weakly expressed Axin‐V5 changes in a biphasic manner (Yang *et al.,* 2016). Initially distributed uniformly in the ectoderm, Axin‐V5 first accumulates in segmental stripes marked by Wg induction. Secondly, with progressing development, Axin levels drop specifically in the Wg stripes, an observation analogous to delayed AXIN destabilization in mammalian cells (Li *et al.,* 2012 and references therein). In embryos expressing an Axin‐V5 mutant variant lacking the TBM or in embryos lacking tankyrase, Axin‐V5 levels are uniformly high. Early Axin‐V5 stripes fail to form, with a concomitant loss of normal Wg target gene expression. Importantly, this is not merely due to increased Axin‐V5 levels under these circumstances since strong Axin‐V5 overexpression does not copy this phenotype (Yang *et al.,* 2016). The authors hence proposed a role of tankyrase in regulating Axin function rather than levels (Figure 1B). Studies in *Drosophila* and work with *Drosophila* cells and HEK293T cells showed that Wg/Wnt stimulation results in accumulation of PARylated Axin‐V5/AXIN1 with an enhanced formation of AXIN‐ and LDL receptor‐related protein 6 (LRP6)/Arrow‐containing Wnt/Wg signalosomes (Yang *et al.,* 2016). Capitalizing on the ability to detect endogenous *Drosophila* Axin, a second study suggests that Axin is present in both the cytoplasm and at the membrane, even under basal conditions. Wg stimulation results in Axin accumulation in both compartments, with an enrichment of PARylated Axin at the membrane (Wang *et al.,* 2016a). Forcing Axin to the membrane under basal conditions increases its PARylation and destabilization, presumably by PARdU, suggesting that tankyrase acts at the membrane or Axin's susceptibility to PARylation is augmented upon membrane recruitment (Wang *et al.,* 2016a). Phosphorylation by GSK3 (Kim *et al.,* 2013) or binding of the small molecule HLY78 (Wang *et al.,* 2013) was reported to induce an open AXIN conformation for productive interaction with LRP6. AXIN PARylation may control the assembly of the Wnt signalosome complex in a similar manner. Alternatively, PAR itself may act as a molecular glue to recruit AXIN to Wnt signalosomes. While the intact LRP5 C‐terminus is required for AXIN binding (Mao *et al.,* 2001), it is presently not clear whether AXIN and LRP5/6 interact directly (Mao *et al.,* 2001; Kim *et al.,* 2013; Wang *et al.,* 2013), but there are indications that this may be the case (MacDonald *et al.,* 2011). Future studies will unravel the mechanisms behind the Wnt/Wg‐induced accumulation of PARylated Axin and the role of PARylation in signalosome assembly. Yang *et al*. (2016) propose that the PAR‐assisted signalosome formation enables a rapid response to Wnt signals while the subsequent down‐regulation of AXIN limits the re‐assembly of β‐catenin destruction complexes, thereby conferring both responsiveness and robustness to the pathway.

## Tankyrase inhibitors

In this section, we provide an overview of TNKSi; for more comprehensive discussions, we refer the reader to other recent reviews (Lehtiö *et al.,* 2013; Steffen *et al.,* 2013; Haikarainen *et al.,* 2014a). All low MW TNKSi developed to date are mimetics of β‐nicotinamide adenine dinucleotide (NAD^+^) or its adenosine or nicotinamide portions. The first potent toolbox TNKSi, XAV939 (Huang *et al.,* 2009), IWR‐1 and IWR‐2 (Chen *et al.,* 2009; Gunaydin *et al.,* 2012), were discovered in phenotypic screens designed to identify antagonists of the Wnt/β‐catenin pathway, as were the inhibitors JW74 (Waaler *et al.,* 2011), JW55 (Waaler *et al.,* 2012), WIKI4 (James *et al.,* 2012) and K‐756 (Okada‐Iwasaki *et al.,* 2016). Numerous additional inhibitors were established through diverse approaches (see Zhan *et al.,* 2014), including screening for compounds that rescue tankyrase‐induced lethality of yeast cells (Yashiroda *et al.,* 2010) or induce a mitotic spindle defect (Johannes *et al.,* 2015b), fragment screening (Larsson *et al.,* 2013; de Vicente *et al.,* 2015), proteomics (Thomson *et al.,* 2017), *in silico* screening or substructure searching, followed by compound optimization (Bregman *et al.,* 2013b; Elliott *et al.,* 2015), screening of a DNA‐encoded library (Samain *et al.,* 2015) and extensive structure–activity relationship studies, assisted by the structural analysis of tankyrase/PARP:inhibitor complexes (Hua *et al.,* 2013; Shultz *et al.,* 2013; Voronkov *et al.,* 2013; Narwal *et al.,* 2013a; Liscio *et al.,* 2014; Qiu *et al.,* 2014; Haikarainen *et al.,* 2014b; Kumpan *et al.,* 2015; Nkizinkiko *et al.,* 2015; Paine *et al.,* 2015; Haikarainen *et al.,* 2016; Thomson *et al.,* 2017). Numerous more drug‐like molecules, with optimized pharmacological properties, are now available, for example, G007‐LK (Lau *et al.,* 2013; Voronkov *et al.,* 2013) and NVP‐TNKS656 (Shultz *et al.,* 2013). Table 1 gives examples of published TNKSi.

### Inhibitor binding sites on the tankyrase PARP domain

**Figure 5:**
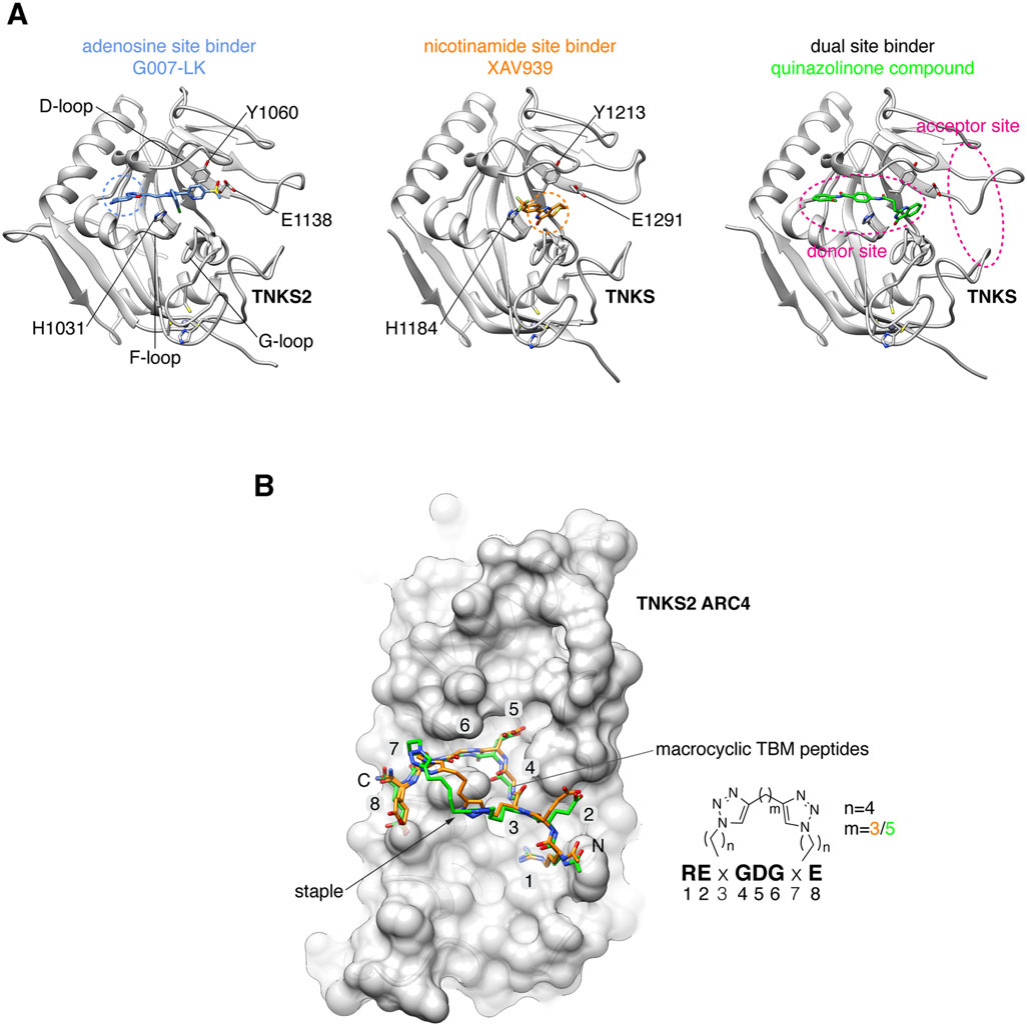
Binding modes of catalytic TNKSi and alternative inhibition strategy. (A) Structural representation of TNKS/TNKS2 PARP domain:inhibitor complexes. Residues of the catalytic H‐Y‐E triad are indicated. Adenosine and nicotinamide sites are highlighted with blue and orange dashed circles, respectively, in the left and central panels, respectively. Left, structure of the TNKS2 PARP domain with the adenosine site binder G007‐LK (shown in blue) [protein data bank (PDB) code 4HYF] (Voronkov *et al.,* 2013). Loops lining the inhibitor binding site are indicated. Centre, structure of the TNKS PARP domain with the nicotinamide site binder XAV939 (shown in orange) (PDB code 3UH4) (Kirby *et al.,* 2012). Right, structure of the TNKS PARP domain with a dual site binder (shown in green) (PDB code 4I9I) (Bregman *et al.,* 2013b). The donor and acceptor sites are highlighted in magenta. (B) Structural representation of TNKS2 ARC4 (in surface representation) bound to two macrocyclized TBM peptides shown in superposition (PDB codes 5BXO and 5BXU) (Xu *et al.,* 2017b). The peptide sequence is shown on the right with the position of the two different peptide staples, whose structures are shown. Amino acid positions of the TBM are indicated.

The tankyrase PARP domain shares strong homology with the catalytic domains of the 17 human Diphtheria‐toxin‐like ADP‐ribosyltransferases (ARTDs) (see Hottiger *et al.,* 2010). Among all ARTDs known to synthesize PAR (Vyas *et al.,* 2014), the TNKS/TNKS2 PARP domains are unique in that they coordinate zinc, lack an N‐terminal helical regulatory subdomain and also lack a loop distal to the active site (Lehtiö *et al.,* 2008). In all other respects, they share the typical structural elements with other ARTD catalytic domains (Figure 5A). A so‐called donor site coordinates the co‐substrate NAD^+^ while the acceptor site accommodates either the peptide for the priming modification or the growing PAR chain poised for extension (Figure 5A). The donor site is lined by three loops: the donor site or D‐loop, a glycine‐rich G‐loop and a phenylalanine‐containing F‐loop (Figure 5A). Tankyrase contains a catalytic H‐Y‐E triad required for PAR synthesis (Figure 5A). All TNKSi developed to date target the NAD^+^‐binding donor site but can broadly be classified by three binding modes, according to our structural understanding of NAD^+^ binding to Diphtheria toxin (Bell and Eisenberg, 1996). Inhibitors either primarily engage the nicotinamide subsite (e.g. XAV939) (Karlberg *et al.,* 2010), the adenosine subsite (e.g. IWR‐1 and G007‐LK) (Narwal *et al.,* 2012; Voronkov *et al.,* 2013) or both sites in the case of the more recently developed dual‐site inhibitors (Bregman *et al.,* 2013b) (Figure 5A; Table 1). In some instances, a phosphate site between the two subsites is specified as well (see Steffen *et al.,* 2013). Crystal structures of tankyrase catalytic domains without inhibitor show closed D‐loop conformations, albeit different ones in apo‐TNKS and apo‐TNKS2 with indications of flexibility (Lehtiö *et al.,* 2008; Karlberg *et al.,* 2010). Adenosine site binders appear to induce their own pocket, conferring disorder or conformational changes to the D‐loop (Lehtiö *et al.,* 2008; Gunaydin *et al.,* 2012; Shultz *et al.,* 2012). Hence, the adenosine subsite appears highly adaptable and is sometimes referred to as the ‘induced pocket’.

### Achieving selectivity

PARP inhibitor profiling against ARTDs revealed a remarkable promiscuity for a number of PARP inhibitors (Wahlberg *et al.,* 2012; Thorsell *et al.,* 2017). For example, XAV939 inhibits TNKS, TNKS2, PARP1 and PARP2 with comparable potency (e.g. IC_50_ values of 95, 5, 74 and 27 nM in a direct comparison using the catalytic domains of TNKS and TNKS2 and full‐length PARP1 and 2, respectively), while IWR‐1 is more specific for the tankyrases (no measurable IC_50_ for full‐length PARPs 1 and 2) (Thorsell *et al.,* 2017). High‐resolution PARP domain:TNKSi co‐crystal structures have (i) rationalized TNKSi selectivity and (ii) enabled structure‐based drug design of more selective and potent tankyrase binders (see Lehtiö *et al.,* 2013; Steffen *et al.,* 2013; Haikarainen *et al.,* 2014a). Specific examples for the former include G007‐LK, which was optimized from JW74 (Lau *et al.,* 2013; Voronkov *et al.,* 2013), and WIKI4 (James *et al.,* 2012; Haikarainen *et al.,* 2013). Examples for the latter include NVP‐TNKS656, developed from XAV939 (Shultz *et al.,* 2013), and dual‐site inhibitors (Hua *et al.,* 2013) (Table 1). These studies demonstrated that the unique structural features of the tankyrase catalytic domain can be exploited to gain selectivity. For example, WIKI4 in the adenosine subsite would sterically clash with the helical subdomain in PARPs 1–3 (Haikarainen *et al.,* 2013). More subtle differences can also be harnessed. Compared to other PARPs, the D‐loop of tankyrases is three amino acids shorter, more flexible and often disordered in crystal structures of the domain with inhibitors, due to the absence of three proline residues, and characterized by large hydrophobic amino acids, which confer a narrower, more hydrophobic donor site pocket (Lehtiö *et al.,* 2008; Wahlberg *et al.,* 2012). Selectivity and potency can be gained by ‘growing’ compounds toward this narrow pocket, as seen in the optimization of quinazolinones (Nathubhai *et al.,* 2013), tetrahydro‐1,6‐naphtyridin‐5‐ones (Kumpan *et al.,* 2015) and the XAV939 core to NVP‐TNKS656 (Shultz *et al.,* 2013) (Table 1). Of note, although selectivity over other ARTDs can be achieved, many other enzymes also use NAD^+^ as a co‐substrate, and so inhibitors designed to target the NAD^+^ donor site may have unknown off‐target effects at high concentrations. However, the example of sirtuins shows that this potential challenge can be overcome (Ekblad and Schüler, 2016).

### Future developments

While further optimization of catalytic TNKSi is progressing, non‐catalytic scaffolding roles of tankyrase in Wnt/β‐catenin signalling are emerging (Mariotti *et al.,* 2016), and these may be augmented when prolonged TNKSi treatment results in tankyrase stabilization by blocked PARdU (Huang *et al.,* 2009). Furthermore, overexpression of tankyrase in several tumour types has been reported (Matsutani *et al.,* 2001; Gelmini *et al.,* 2004, 2006, 2007; Shervington *et al.,* 2007; Shebzukhov *et al.,* 2008; Zhao *et al.,* 2009; Gao *et al.,* 2011; Tang *et al.,* 2012; Busch *et al.,* 2013) and may accentuate tankyrase's concentration‐dependent scaffolding functions, contributing to TNKSi resistance (Mariotti *et al.,* 2016). Therefore, blocking tankyrase's ARC‐ and SAM‐dependent scaffolding functions holds considerable potential. Importantly, the ARCs and SAM domain are highly conserved between the two tankyrases (Figure 2A) but unique among the ARTD family, therefore offering the opportunity for target selectivity of potential compounds over other ARTDs, in addition to the potential benefits of inhibiting non‐catalytic scaffolding functions. Moreover, potential interference with other NAD^+^‐dependent enzymes could be circumvented by this approach. Whereas blocking SAM‐domain‐dependent polymerization appears challenging due to the relatively shallow polymerization interface, targeting the deeper TBM‐binding pocket on the ARCs is more promising (Guettler *et al.,* 2011; Morrone *et al.,* 2012). This binding pocket is not conserved across ankyrin repeat proteins in general and appears to be unique to tankyrase. Given the presence of four substrate‐binding ARCs, blockage of each of these substrate/ligand binding sites is likely to be required, in both TNKS and TNKS2. However, this appears feasible given the conservation of the TBM‐binding pocket across the TNKS and TNKS2 ARCs (Guettler *et al.,* 2011). Xu *et al*. (2017b) have recently shown that a stapled TBM peptide, based on a previously reported optimized TBM sequence (Guettler *et al.,* 2011) and fused to a cell‐permeability conferring peptide, can compete with AXIN and block Wnt/β‐catenin signalling (Figure 5B). This proof‐of‐concept study will encourage further development of tankyrase substrate binding antagonists. While a recent tankyrase interactome study is in agreement with the notion that TNKS and TNKS2 are largely functionally redundant (Li *et al.,* 2017), there may be benefit to selectively targeting either TNKS or TNKS2, should unique functions emerge in the future, which may be TNKS/TNKS2‐intrinsic or result from other sources such as differential expression or regulation. One inhibitor study (see Table 1, compound **3**) suggests that, in principle, a certain degree of such selectivity can be achieved (Larsson *et al.,* 2013).

## Functional and preclinical studies of tankyrase inhibitors in CRC

### Differential sensitivity of CRC cell lines to tankyrase inhibition

**Figure 6:**
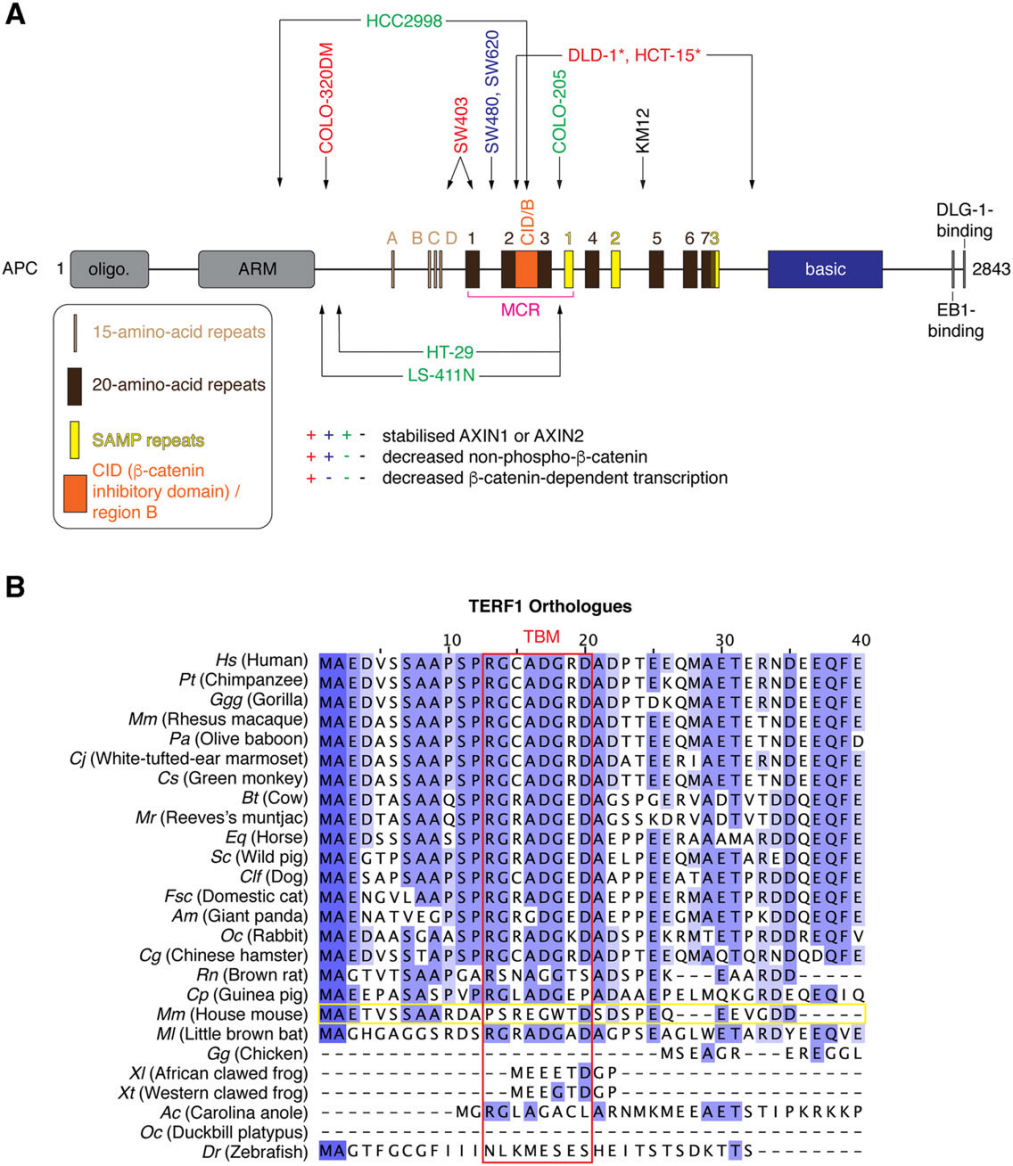
Potential determinants of TNKSi responses: APC mutation status and telomeric roles of tankyrase. (A) Schematic representation of APC with domains and motifs drawn to scale (see Stamos and Weis, 2013). So‐called 15‐ and 20‐amino‐acid‐repeats (15R and 20R) bind β‐catenin (except for 20R2), with the affinity of the 20Rs for β‐catenin being enhanced by their phosphorylation (Eklof Spink *et al.,* 2001; Ha *et al.,* 2004; Xing *et al.,* 2004). SAMP repeats bind to AXIN1/2 (Spink *et al.,* 2000). 20R2 and the catenin interaction domain (CID) / region B are required for β‐catenin ubiquitylation but do not bind β‐catenin; instead, they regulate AXIN1/2 binding to APC, and CID / region B is proposed to bind α‐catenin (Liu *et al.,* 2006; Kohler *et al.,* 2008; Choi *et al.,* 2013; Pronobis *et al.,* 2015). The mutation cluster region (MCR), a mutation hotspot in CRC (Kohler *et al.,* 2008), is indicated in magenta. APC truncations observed in commonly used CRC cell lines are indicated by the arrows (Rowan *et al.,* 2000; Ikediobi *et al.,* 2006); labels are colour‐coded according to the indicated effects of TNKSi on AXIN and non‐phospho (active) β‐catenin levels and β‐catenin‐dependent transcription. *Note that the classification of DLD‐1 and HCT‐15 cells as TNKSi‐sensitive or ‐resistant varies between studies, given an ‘intermediate’ response (Huang *et al.,* 2009; Lau *et al.,* 2013; de la Roche *et al.,* 2014; Tanaka *et al.,* 2017). Very low AXIN1/2 levels in KM12 cells (Tanaka *et al.,* 2017) may be responsible for non‐detectable AXIN accumulation upon tankyrase inhibition. (B) Multiple sequence alignment of the N‐termini of TERF1/TRF1 (telomeric repeat binding factor 1) orthologues from the indicated species, coloured by percentage identity. The amino acid numbering refers to human TERF1. The 8‐amino‐acid TBM is boxed in red. The murine Terf1 orthologue sequence is boxed in yellow and shows no conservation of the TBM.

Most CRC tumours (≈80%) are hemizygous for C‐terminal truncations of APC (see Bodmer, 2006), focussed at a hotspot area known as the mutation cluster region (Miyoshi *et al.,* 1992; Kohler *et al.,* 2008; see Minde *et al.,* 2011) (Figure 6A). Following the N‐terminal Armadillo repeat domain, APC contains four 15‐amino‐acid β‐catenin‐binding repeats, seven 20‐amino‐acid β‐catenin‐binding repeats and three interspersed AXIN‐binding SAMP repeats, among other elements not required for APC's function in Wnt/β‐catenin signalling (see Stamos and Weis, 2013). Dependent on the position of the truncating mutation, a variable number of these motifs is lost (Figure 6A). In APC‐truncated CRC cells, an inability to assemble a functional β‐catenin destruction complex underlies the accumulation of transcriptionally active β‐catenin. A range of studies has investigated the responsiveness of model CRC cell lines to tankyrase inhibition (Lau *et al.,* 2013; de la Roche *et al.,* 2014; Tanaka *et al.,* 2017). While it would have been conceivable that the assembly and function of the β‐catenin destruction complex cannot be sufficiently rescued by tankyrase inhibition in the absence of fully functional APC, there clearly are cases in which dysregulated Wnt/β‐catenin signalling can be curbed. In SW403 and COLO‐320DM cells, both of which bear extensive C‐terminal APC truncations (Figure 6A), tankyrase inhibition (by G007‐LK, IWR‐1 or XAV939) gives rise to AXIN2 stabilization, the formation of β‐catenin degradasomes (in COLO‐320DM cells), a robust reduction in active (non‐phosphorylated) β‐catenin and prominently attenuated β‐catenin‐dependent transcription, both in reporter assays and at the level of endogenous Wnt/β‐catenin target genes (Lau *et al.,* 2013; de la Roche *et al.,* 2014; Tanaka *et al.,* 2017). Importantly, tankyrase inhibition limits the proliferation of these cells in cell culture (for G007‐LK and IWR‐1) and xenograft (for G007‐LK) models (Lau *et al.,* 2013; Tanaka *et al.,* 2017). A similar cell response to tankyrase inhibition is observed in DLD‐1 and HCT‐15 cells, although the levels of active β‐catenin are reduced less robustly and the transcriptional effect is more subtle (Lau *et al.,* 2013; de la Roche *et al.,* 2014; Tanaka *et al.,* 2017). Conversely, in SW480 and SW620 cells, although tankyrase inhibition results in the stabilization of AXIN2, degradasome formation and a strong decrease in active β‐catenin levels, it fails to block Wnt/β‐catenin target genes or the TOPFlash reporter (Lau *et al.,* 2013; de la Roche *et al.,* 2014). In yet another group of CRC cells (COLO‐205, HT‐29, HCC2998 and LS‐411 N), tankyrase inhibition increases AXIN1/2 levels but with only a modest or no decrease in active β‐catenin levels (Lau *et al.,* 2013; Tanaka *et al.,* 2017). For KM12 cells, no effect on the levels of AXIN1/2 or active β‐catenin levels and β‐catenin‐dependent transcription was observed upon tankyrase inhibition (Tanaka *et al.,* 2017). As expected, Wnt/β‐catenin signalling in CRC cells with oncogenic mutations in β‐catenin (LS174T, HCT116) or β‐catenin‐independent CRC cells (RKO) are not sensitive to tankyrase inhibition (Lau *et al.,* 2013; Tanaka *et al.,* 2017).

Tankyrase inhibition can therefore restore, at least partly, β‐catenin destruction complex function in a subset of APC‐mutant CRC cells (Figure 6A). Why another subset of CRC cell lines is TNKSi‐resistant is being investigated. It has been proposed that high levels of β‐catenin in SW480 cells account for TNKSi resistance (Lau *et al.,* 2013), but β‐catenin levels in the TNKSi‐sensitive COLO‐320DM cells appear comparable (de la Roche *et al.,* 2014). Compared to SW480, COLO‐320DM cells appear to have higher levels of AXIN1, TNKS and phospho‐β‐catenin after tankyrase inhibition (de la Roche *et al.,* 2014), suggesting that β‐catenin may be sequestered in stalled destruction complexes, thereby limiting the availability of active β‐catenin.

A potential correlation between the site of APC truncation and TNKSi sensitivity has been explored, both in CRC cell lines and tumour‐derived cells from patients (Tanaka *et al.,* 2017). APC truncations removing all β‐catenin‐binding 20‐amino‐acid repeats (as in COLO‐320DM and SW403 cells; Figure 6A) were proposed to render cells TNKSi‐responsive at the level of cell proliferation and might serve as a predictive biomarker (Tanaka *et al.,* 2017). Another distinguishing feature of these cells is their particularly strong Wnt/β‐catenin pathway activity (Tanaka *et al.,* 2017). The authors suggest that the longer APC variants of other cell lines act as hypomorphs maintaining a higher residual level of β‐catenin regulation: their silencing further stabilizes β‐catenin. TNKSi (G007‐LK, IWR‐1) can reverse this accumulation but not reduce β‐catenin abundance below its cell‐characteristic elevated levels (Tanaka *et al.,* 2017). In turn, cell proliferation remains unresponsive to tankyrase inhibition (Tanaka *et al.,* 2017). The genetic background of additional cell lines and tumour samples will need to be explored to confirm the suitability of APC truncations as predictive biomarkers for TNKSi sensitivity. In line with a requirement of AXIN2 for degradasome formation (see above), TNKSi depend on AXIN2 to reduce active β‐catenin levels (Tanaka *et al.,* 2017). Large APC truncations, eliciting high β‐catenin activity and thus *AXIN2* gene transcription, may be required for the TNKSi‐induced accumulation of sufficient amounts of AXIN2. Indeed, absolute *AXIN2* mRNA levels are high in COLO‐320DM cells and, upon tankyrase inhibition, remain higher than in many other CRC cell lines (Tanaka *et al.,* 2017).

Upon prolonged Wnt stimulation, sequestration of β‐catenin in nuclear transcriptional complexes may shield β‐catenin from the β‐catenin destruction complex and account for TNKSi (XAV939) resistance (de la Roche *et al.,* 2014). High expression levels of lymphoid enhancer‐binding factor 1 (LEF1) and B9L and a CRC environment providing sustained Wnt levels are potential predictors of TNKSi resistance (de la Roche *et al.,* 2014). Moreover, the acquisition of APC mutations is considered an early event in the emergence of CRC, and secondary mutations in genes such as *KRAS*, *P53* and *SMAD4* contribute to driving carcinogenesis (Drost *et al.,* 2015); such mutations may modulate the TNKSi response, although this remains speculative. Of note, DLD‐1 colony formation can be inhibited with XAV939 under low‐ but not high‐serum conditions (Huang *et al.,* 2009; Bao *et al.,* 2012; Lau *et al.,* 2013), and similarly, DLD‐1 and HCT‐15 colony formation does not respond to G007‐LK at high serum, while that of COLO‐320DM and SW403 cells does (Lau *et al.,* 2013). This points to additional sensitivity determinants outside the APC and β‐catenin mutational landscape, although simple compound sequestration by serum components may in some cases also contribute. In support of a more complex determination of sensitivity, Mashima *et al*. (2017) generated a TNKSi‐resistant COLO‐320DM line, showing decreased Wnt/β‐catenin signalling and up‐regulated mTOR signalling. These cells were generated to tolerate IWR‐1 but also displayed considerable resistance to G007‐LK. The authors showed that the mTOR pathway determines TNKSi resistance in these cells. In conclusion, we need to better understand how the genetic and signalling profile of CRC cells and tumours affects TNKSi responsiveness. Furthermore, a much deeper analysis of the β‐catenin destruction complex and the Wnt signalosome is required to appreciate the mechanisms by which tankyrase inhibition affects the molecular events underlying Wnt/β‐catenin signalling, both in the context of wild‐type and mutant APC.

### Tankyrase inhibitors in murine models

*In vivo* preclinical studies have demonstrated the anti‐tumour activity of various TNKSi (Waaler *et al.,* 2011, 2012; Lau *et al.,* 2013). JW74 was studied in both xenograft and the Apc^Min^ mouse models and reported to be well tolerated while reducing both the total tumour load in the small intestine and the tumour number in the colon (Waaler *et al.,* 2011). Similar observations were made for JW55 in mice with a conditional Apc truncation in the ISC compartment (Waaler *et al.,* 2012). An improved derivative of JW74, G007‐LK, which inhibits tankyrase with double‐digit nanomolar IC_50_ values and good specificity (Table 1) (Voronkov *et al.,* 2013), decreases the tumour area in the small intestine of these mice by approximately two thirds and shows significant inhibition of tumour growth in various xenograft models with APC‐mutant human cell lines (SW480, COLO‐320DM and SW403) (Lau *et al.,* 2013).

Intestinal toxicity remains a major challenge for many Wnt/β‐catenin pathway inhibitors (see Kahn, 2014). A more careful analysis of the effects of G007‐LK at dose‐limiting levels revealed reduced cell proliferation in crypt bases of the small intestine, inflammation, necrosis, disrupted epithelial architecture, with ensuing weight loss and moribundity (Lau *et al.,* 2013). Whether the observed toxicity is reversible has not been explored. Conversely, in a study investigating the role of tankyrase in glucose metabolism, the long‐term (6 months) treatment of mice with G007‐LK at a lower dose delivered orally, as opposed to intraperitoneally, did not result in detectable toxicity despite the observed stabilization of Axin1 and reduction in active β‐catenin levels (Zhong *et al.,* 2016a). As the expression of the Wnt pathway antagonist Dickkopf‐related protein 1 (Dkk1) in the gut epithelium gives rise to similar toxicity as highly dosed G007‐LK (Pinto *et al.,* 2003; Kuhnert *et al.,* 2004; Lau *et al.,* 2013), it is likely that TNKSi toxicity is an on‐target, Wnt/β‐catenin pathway‐specific effect. A mouse xenograft study evaluating the TNKSi G‐631 (patent by Feng *et al.,* 2013) also revealed considerable intestinal toxicity, even at sub‐therapeutic doses (Zhong *et al.,* 2016b). Importantly, ablation of Wnt signalling by either adenoviral Dkk1 expression or treatment with G‐631 is reversible (Kuhnert *et al.,* 2004; Zhong *et al.,* 2016b).

While toxicity poses challenges in continued efforts to explore the therapeutic potential of TNKSi, it is no reason to be discouraged. Does TNKSi toxicity indeed reflect on‐target or off‐target action of the inhibitors? What is the tissue distribution of the compounds? For example, do they accumulate in the gastrointestinal tract, aggravating toxicity? Do chemically distinct TNKSi display similar signs of toxicity? The fact that SW480 cell xenograft growth can be contained by tankyrase inhibition despite the incomplete penetrance on β‐catenin dependent transcription (see above) suggests that full inhibition of signalling might not be required for a therapeutic effect; so what degree of β‐catenin inhibition translates into a biological effect? Moreover, different dosage and timing regimes could be implemented to manage known toxicities (see Meric‐Bernstam and Mills, 2012). Encouragingly, inhibitors of the acyl transferase Porcupine, which palimitoylates Wnt during its biogenesis, show limited intestinal toxicity at effective doses, suggesting that substantial therapeutic windows can be achieved by targeting the Wnt/β‐catenin pathway (Liu *et al.,* 2013; Proffitt *et al.,* 2013; see also Madan and Virshup, 2015). The point of intervention in the pathway, drug specificity, potency and method of delivery, pharmacokinetics, functional redundancy of targeted pathway components and the genetic background of the tumour cells may all define the therapeutic window. Combination with inhibitors targeting additional cancer dependencies (e.g. EGFR and PI3K‐AKT) provides another possible strategy for increasing the effectiveness of TNKSi while ensuring their safety (Casas‐Selves *et al.,* 2012; Tenbaum *et al.,* 2012; Arques *et al.,* 2016).

A deeper knowledge of the responses and toxicities elicited by Wnt/β‐catenin pathway modulators in different species is much needed to exploit β‐catenin dependencies in cancer. With the vast range of known and putative tankyrase targets (Guettler *et al.,* 2011; Li *et al.,* 2017), it would be surprising if TNKSi effects and toxicities were entirely due to Wnt/β‐catenin pathway inhibition. Another prominent system highly relevant to the human stem cell compartment is telomere length homeostasis, which is also regulated by tankyrase, as is sister telomere resolution in mitosis (Smith *et al.,* 1998; Smith and de Lange, 2000; Canudas *et al.,* 2007; Kulak *et al.,* 2015). Telomeric functions require tankyrase to bind telomeric repeat‐binding factor 1 (TRF1/TERF1). Importantly, telomere regulation by tankyrase is not conserved in mice since murine Trf1/Terf1 lacks the TBM (Figure 6B) and does not bind tankyrase (Muramatsu *et al.,* 2007; see Hsiao and Smith, 2008). Consistent with the absence of a role for telomeric tankyrase functions in mice, Tnks and Tnks2 knockout mice do not display any telomere phenotype (Hsiao *et al.,* 2006; Chiang *et al.,* 2008), but a definitive answer will be obtained from comparing murine and human cells deficient in both tankyrases. Therefore, preclinical studies of TNKSi in mice and other species lacking the Terf1 TBM (e.g. zebrafish; Figure 6B) are unlikely to predict the full extent of biological effects and toxicity in humans. Conversely, rabbits or Chinese hamsters, for example, both display a functional TBM in Terf1 and might be more suitable models for studying the *in vivo* consequences of tankyrase inhibition, at least with regards to Wnt/β‐catenin signalling and telomere maintenance. In rats, the somewhat stronger deviation of the TBM will require a prior validation of a telomeric role for tankyrase (Muramatsu *et al.,* 2007). Telomere maintenance in *Drosophila* occurs *via* a transposon‐mediated mechanism rather than telomerase (Villasante *et al.,* 2008), and a telomeric tankyrase link in flies is therefore unlikely. While Wg/Armadillo pathway regulation by tankyrase is clearly evident in *Drosophila*, some mechanistic aspects may be different, for example given that *Drosophila* Axin only bears a single TBM (Figure 2C).

## Outstanding questions

The past few years have seen a rapid progress in our understanding of how Wnt/β‐catenin signalling is regulated by PARylation and tankyrase. Tankyrase is now an established core component of the Wnt/β‐catenin network. Nonetheless, we are still far from a full understanding of the complex roles that tankyrase plays in the pathway. How does tankyrase promote Wnt/β‐catenin signalling non‐catalytically, and do these mechanisms contribute to TNKSi resistance? Does scaffolding through tankyrase directly control β‐catenin degradasome assembly, and how is this process regulated? How does AXIN PARylation promote the function of the Wnt signalosome? It will be interesting to explore the consequences of AXIN PARylation on both its conformation and interactions with components of both the signalosome and degradasome complexes. Given early indications of a role for tankyrase in APC‐regulated destabilization of AXIN, additional work is needed to decipher how APC limits AXIN abundance. TNKSi have now reached a remarkable specificity. The continued exploration of their pharmacodynamics, the identification of potential biomarkers for their therapeutic implementation and the in‐depth analysis of emerging resistance and toxicity mechanisms are important avenues of further research. The latter will require a careful choice of model systems. The development of alternative tankyrase inhibition strategies through interfering with the non‐catalytic, scaffolding functions of tankyrase, in particular substrate binding, will undoubtedly offer new and exciting opportunities to understand tankyrase function and explore alternative therapeutic strategies.

### Nomenclature of targets and ligands

Key protein targets and ligands in this article are hyperlinked to corresponding entries in http://www.guidetopharmacology.org, the common portal for data from the IUPHAR/BPS Guide to PHARMACOLOGY (Southan *et al.,* 2016), and are permanently archived in the Concise Guide to PHARMACOLOGY 2015/16 (Alexander *et al.,* 2015a, 2015b).

## Conflicts of interest

The authors declare no conflicts of interest.
